# The Occurrence of Non-handaxe Assemblages Early in the Purfleet Interglacial (MIS 9) in Britain

**DOI:** 10.1007/s41982-025-00217-2

**Published:** 2025-05-17

**Authors:** Aaron Rawlinson, Rob Davis, Nick Ashton, David Bridgland, Luke Dale, Simon Lewis, Mark White

**Affiliations:** 1 Department of Britain, Europe and Prehistory, British Museum, 38 - 56 Orsman Road, London, N1 5QJ UK; 2 Department of Geography, Durham University, Durham, England DH1 3LE UK; 3 Department of Archaeology, Durham University, Durham, England DH1 3LE UK; 4 School of Geography, Queen Mary University of London, Mile End Road, London, E1 4 NS UK

**Keywords:** Clactonian, Acheulean, Britain, MIS 9, Purfleet, Redhill

## Abstract

Despite the widespread acceptance of the Clactonian during the earlier part of the Hoxnian interglacial (MIS 11c) in Britain, the subsequent occurrence of a non-handaxe signature early in the following interglacial (MIS 10/9) has received less coverage and remains contentious. Recent work on MIS 9 in Britain has re-evaluated the archaeology of the period. This paper offers a critical assessment of the non-handaxe signature in MIS 9 Britain, including the addition of Redhill in East Anglia from recent excavations. The paper is an evaluation of the evidence for distinct core and flake assemblages, analysing the technology and offering a comparison with handaxe sites from the interglacial. Four non-handaxe sites can be identified in the Thames and East Anglia; the technology of the artefacts they yield represents a base line that cannot be differentiated from the wider Lower Palaeolithic other than in terms of the presence/absence of handaxe manufacture. Due to the lack of positive identifiers, the MIS 9 technology cannot be linked directly to the Clactonian and should be treated separately. Given the temporal pattern of occurrence early in the interglacial in both MIS 11 and MIS 9, and no convincing functional or raw-material explanations, the assemblages are interpreted as a cultural signature. This fits into the wider variation across Europe during the Lower Palaeolithic linked to the ‘Cultural Mosaic Model’, and a further example of the nuanced chronological patterns emerging in the Lower Palaeolithic of Britain.

**Supplementary Information:**

The online version contains supplementary material available at 10.1007/s41982-025-00217-2.

## Introduction

**Fig. 1:**
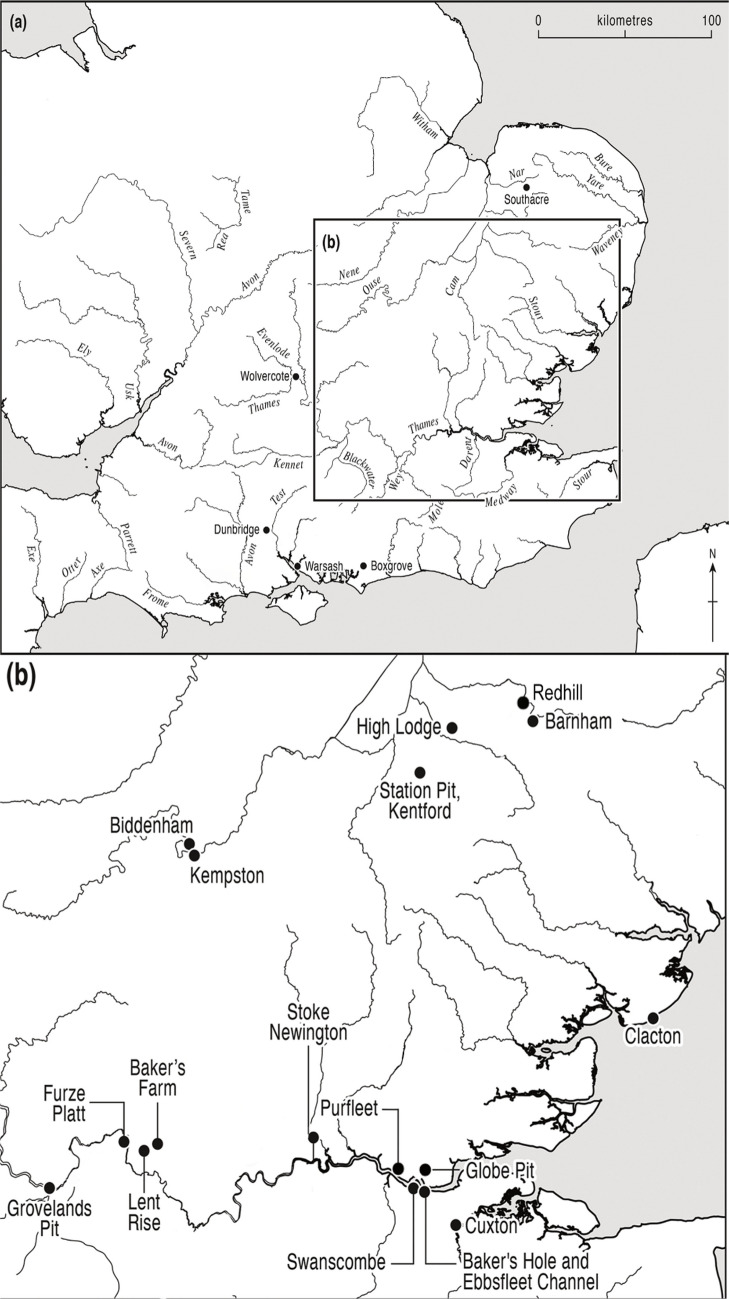
Map of key Lower Palaeolithic sites discussed in the text. **a** Map of Britain. **b** Inset of Thames Valley and East Anglia (after Rawlinson et al., 2022)

Recent work on the ‘Purfleet Interglacial’, inclusive of the terminal warming and cooling transitions from juxtaposed glacial episodes (Marine Isotope Stages 10–9–8), has begun challenging previously held ideas about the technology of the late Lower Palaeolithic (White & Bridgland, 2018; Rawlinson, 2021; Dale, 2022; Rawlinson et al., 2022; Dale et al., 2024; White et al., 2024). Previous work posited a tripartite sequence of Clactonian, Acheulean and ‘Proto-Levallois’/Levallois assemblages during the interglacial, as suggested by work at Purfleet (Palmer, 1975; Wymer, 1985; Schreve et al., 2002; Bridgland et al., 2013; SOM1 Table 1). However, critical re-evaluations of indicators of early Middle Palaeolithic behaviour such as the purported increase in the number of flake tools (Rawlinson et al., 2022) and simple prepared cores (SPC)/proto-Levallois technology (White et al., 2024) has questioned the later part of this tripartite sequence, demonstrating that rather than showing an embryonic Middle Palaeolithic, the assemblages from MIS 9 in Britain are more characteristic of variation within the Lower Palaeolithic. It is therefore timely to take a fresh look at the evidence for the non-handaxe sites that have been argued to be characteristic of MIS 10/9 (Fig. 1).

### The Clactonian

In Britain, non-handaxe assemblages dated to the Middle Pleistocene have traditionally been classified as ‘Clactonian’ after the type-site of Clacton-on-Sea, Essex, where Kenworthy (1898) and Warren (1911, 1912) first recognised industries entirely composed of cores and flakes and lacking handaxes. Warren (1926) first used the term Clactonian in a footnote, describing the industry as a parallel culture to the Acheulean, and predecessor to the Mousterian.

The main sites assigned to the Clactonian, due to the absence of evidence for handaxe manufacture, were Clacton and Globe Pit (Little Thurrock), as well as lower stratigraphic levels at Swanscombe (Lower Gravel and Lower Loam) and at Barnham (Gravel Beds) (Wymer, 1968). These were interpreted as representing the first occupation of Britain, preceding the Acheulean (Wymer, 1968, 1974; Collins, 1969). In order to bolster the largely negative definition, many workers sought more positive identifiers for the Clactonian, including the presence of chopper cores, specific types of flake tools such as notches, and large thick flakes with prominent bulbs of percussion and obtuse angles (Table 1). Additionally, the working of cores was often not seen as part of the Acheulean repertoire and therefore, for much of the twentieth century, sites containing an abundance of flakes and cores were often referred to as having ‘Clactonian components’ (Smith, 1933; King & Oakley, 1936; Lacaille, 1940; Paterson & Fagg, 1940; Wymer, 1957, 1968). An unintended consequence of this meant that by the 1960s, the term Clactonian was being used to describe both a culture (the Clactonian) and a technique (Clactonian flaking and its products), and had extended its reach from SE England to the whole globe.

**Table 1:** Clactonian typologies (after White, 2000; Pettitt & White, 2012; Rawlinson, 2021)

Archaeologist	Typology
Warren (1922, 1923, 1924)	Flakes—large and trimmed Cores—discoidal cores and choppers Tools—pointed implements
Chandler (1929, 1935)	Flakes—large, obtuse angle, prominent bulb of percussion (sometimes two), unfaceted, thick and wide, rare secondary working Cores—potential chopper cores, large. Crude handaxes or tortoise cores Use of anvil stones with bruised edges Flake tools—strepy points
Oakley and Leakey (1937)	Flakes—similar to Chandler, also notes use of bold flaking Cores—seen as a waste product dedicated to producing flake tools, potentially utilised as a secondary purpose. Tortoise core element-knapping only on one side to use as a chopper Flake tools—identifies many tool types including nosed scrapers, trilobed hollow scrapers, discoidal scrapers, triangular points, beaked points and butt end scrapers Handaxes not completely absent
Paterson (1937)	Seen as part of an evolutionary scheme, and more of a technical term than a culture Flakes—struck on an anvil, big bulbs, conical, multiple strikes with shattered butts Cores—including choppers and core tools Flake tools—points, side scrapers, notches, nosed scrapers
Warren (1951)	Flakes—broad platform, strong bulb, low flaking angle Cores—some minimally exploited. Anvil stones Core tools—pointed nodule tools, choppers, axe edged tool, discoidal forms. (Some of these could grade into crude handaxes) Flake tools—side scrapers, bill-hook forms, endscrapers, bulb-scrapers, sub-crescent forms, proto-Mousterian points and notches
Wymer (1968, 1974)	Flakes (same as Warren, 1951) Cores—pebble chopper cores, bi-conical chopper cores, proto-handaxe cores Flake tools—non-standardised

For some researchers, this was seen as an overextension of the Clactonian, and there were questions concerning the status of the Clactonian as cultural entity separate from the Acheulean (McNabb & Ashton, 1992; Ashton & McNabb, 1992; Ashton et al., 1994). Research demonstrated that the Clactonian was not primitive or lacking in skill, but showed similar core reduction methods to the Acheulean (Ohel, 1979; McNabb, 1992, 1996), and that there was little that was culturally distinct from Acheulean assemblages, as several of the Clactonian assemblages contained the occasional handaxe (McNabb & Ashton, 1992). Finally, the adoption of the MIS framework and the discovery of Boxgrove (and attribution of other handaxe contexts to MIS 13, such as High Lodge (Ashton et al., 1992), proved the appearance of a refined Acheulean predating evidence of the Clactonian (Shotton et al., 1993; Wenban-Smith, 1999; Austin et al., 1999; Pope, 2002; Keen et al., 2006; Pope et al., 2020). These factors contributed to the concern that the definition of the Clactonian was based solely on an absence of evidence (Ashton et al., 2005).

Despite this new evidence, some defended the importance of the Clactonian as a distinct industry (Wenban-Smith, 1998; White, 2000). Support for the Clactonian rested on whether chronological separation between Clactonian and handaxe assemblages could be demonstrated (Ashton et al., 2005). Continued work on the chronology of the British Lower Palaeolithic (Bridgland, 1994; Schreve, 1997, 2001a, b; Candy & Schreve, 2007; Penkman et al., 2013; Bridgland & White, 2014, 2015; White & Bridgland, 2018), re-evaluation of the handaxes from Clactonian contexts (White, 2000; Pettitt & White, 2012) and new evidence from Barnham (Ashton et al., 2016) and Ebbsfleet (Wenban-Smith et al., 2006; Wenban-Smith, 2013) have meant the Clactonian has been re-evaluated and its definition updated in line with the current evidence. Clactonian sites can now be dated to the earlier part of the Hoxnian interglacial (MIS 11c) up to and including Hoxnian pollen zone II (HoII) (Ashton et al., 2016), preceding the Acheulean industries in the later part of the Hoxnian (Ho III-IV). The Clactonian is recognised stratigraphically below Acheulean layers at Barnham East Farm, Swanscombe and the Elephant Butchery Site in the Ebbsfleet Valley, which can be correlated with the assemblages at Clacton, all of which are Clactonian.

What the work of the 1980s–1990s has done is strip away much of the baggage of the previous decades and left a temporally constrained period during which hominins without handaxe manufacture as part of their cultural repertoire visited Britain, thus satisfying Ashton et al.’s (2005) requirement for a clear chronological separation. Whist debate on the Clactonian continues, this tends to concern why it occurred rather than if it occurred, with even the most vehement critics of the Clactonian now acknowledging its existence (Ashton et al., 2016; McNabb, 2020).

This paper cannot cover over a century of debate over the explanation for the Clactonian, the discussion of which has been provided by McNabb (2007, 2020) and White (2000, 2023), but is predicated on the fact that the Clactonian is represented at several sites representing the earlier part of the Hoxnian interglacial (MIS 11c) that show no evidence of handaxe manufacture (White & Schreve, 2000; Ashton et al., 2016). This chronological pattern and the local geology of the sites concerned also means that previous explanations for the lack of handaxes, namely raw material (Oakley & Leakey, 1937; Ohel, 1979; Singer et al., 1973), function (Rolland, 1992; Bosinski, 1995; Sharon & Barsky, 2016) or a preparatory stage in manufacture (Ohel, 1979; Ohel & Lechevalier, 1979), fail to pass muster.

Whereas it has been suggested that the Clactonian could be the result of distinct hominin species (Stringer, 2011, 2012; Manzi, 2016), Dennell et al. (2011) argued for different hominin 'demes' being present in Europe during the Middle Pleistocene that distinguished between the concept of separate populations from strict differences at a species level. They suggested that there was an ebb and flow of populations with ‘source’ areas in the south of Europe (Iberia, Italy and the Balkans) and ‘sink’ areas in the north and east, which were subject to local extinction. Arrivals from outside of Europe also acted as a source of influx of genetic and phenotypic variability for Europe. As northern Europe was being re-colonised multiple times, potentially from different routes, Dennell et al. (2011) argued that varying traditions, such as the Acheulean and Clactonian, were to be expected. Dennell et al.’s (2011) model helps explain the non-linear Lower Palaeolithic record in Britain. Recently, Ashton and Davis (2021) have proposed the ‘Cultural Mosaics Model’ to explain variation in Lower Palaeolithic technology in Europe based on the idea of small-scale cultural groupings creating localised traditions with distinctive material culture, in part in response to the local environment.

### The return of the Clactonian?

Of the traditional Clactonian sites, Globe Pit (Little Thurrock) has remained an outlier dating to late MIS 10/early 9 rather than MIS 11 (Bridgland & Harding, 1993; Bridgland, 1994; White, 2000). Previously, the similarities between Globe Pit (Little Thurrock) and the MIS 11 Clactonian sites prompted the invention of convoluted terrace formation schemes designed to argue that the deposits around the Grays-Thurrock area were broadly contemporary to the Lower Gravel/Loam at Swanscombe (King & Oakley, 1936). Further work has shown these deposits to date to MIS 10–9 - 8 (Bridgland, 1994; Schreve et al., 2002; Bridgland et al., 2013).

Despite the re-evaluation of the Clactonian, the occurrence of non-handaxe assemblages dating from the following interglacial has not received the same attention due to the lack of primary-context sites representing MIS 9 and the smaller assemblage sizes (McNabb, 2007, 2020; Wenban-Smith, 2013; White & Bridgland, 2018). The MIS 9 non-handaxe signature has been overlooked in comparison to the well debated Clactonian of MIS 11, but it may be crucial to understanding non-handaxe assemblages in Britain, and Europe more widely. We resist using the label Clactonian to describe the non-handaxe sites in MIS 9 as there is no evidence of a direct phylogenetic link between the two occurrences. Instead, the evidence is best treated as a separate phenomenon.

There is a paucity of recently excavated assemblages attributable to MIS 9 that have good levels of preservation. There are just five well-studied ‘flagship’ sites (after Gamble, 1996) from the Lynch Hill - Corbets Tey Formation of the Thames and its equivalents in the River Medway, which offer the best understanding of MIS 9 archaeology (SOM1 Table 2). Three of these sites have been argued to contain non-handaxe assemblages (White, 2000; White & Schreve, 2000; White & Bridgland, 2018). The key site of Purfleet preserves three fining-upward fluvial cycles, the basal cycle (beds 1–3) having been correlated with late MIS 10 / early MIS 9, based on lithological and biostratigraphic evidence (Schreve et al., 2002; Bridgland et al., 2013). The Little Thurrock Gravel at Globe Pit represents an equivalent to Purfleet Beds 1–3 and similarly forms a basal part of the Lynch Hill-Corbets Tey Formation (Bridgland & Harding, 1993). Despite lacking faunal evidence, the Little Thurrock Gravel can be correlated with the basal gravels at Purfleet and is stratigraphically beneath the ‘Grays Brickearth’, which has been suggested as a biostratigraphical type locality for MIS 9 (Schreve, 1997, 2001a, b). Cuxton, in the Medway, a south-bank tributary of the Thames, has been stratigraphically correlated with these sites (Bridgland, 2006) and an assemblage from the lower layers at the site (layers 1–6) yielded a non-handaxe assemblage. Therefore, the three sites of Purfleet, Globe Pit and Cuxton have been argued to represent a time-constrained period during late MIS 10 / early 9 during which non-handaxe-making populations were in Britain, similar to the Clactonian of early MIS 11 (White & Schreve, 2000).

Both Purfleet and Cuxton record the occurrence of handaxe-making populations later in the interglacial. The handaxes from MIS 9 are often characterised by the presence of pointed forms and the co-occurrence of ficrons and cleavers (referred to as Roe’s (1968b) Group I), as demonstrated at Cuxton and the fourth flagship site: Stoke Newington (White et al., 2018; Dale, 2022). In contrast, the final flagship site of Wolvercote presents a different modal type (Roe’s Group III) characterised by ‘slipper shaped’ plano-convex handaxes (Tyldesley, 1986). It has previously been argued that there is evidence  from MIS 9 for the in situ development of Levallois technology via a proto-Levallois stage, most prominently from the higher beds (6–8) in the Purfleet sequence, especially at Botany Pit (White & Ashton, 2003; Scott, 2011; Bridgland et al., 2013), although this has recently been questioned by White et al. (2024).

There is also a number of secondary-context sites in the Thames (SOM1 Table 3), all with historic collections from river terrace aggradations attributed to MIS 10–9–8 (Bridgland, 1994, 2006). These show similar patterns to the flagship sites, in terms of handaxe form, flake tools and cores (White & Bridgland, 2018; Rawlinson, 2021; Davis et al., 2021, 2024; Dale, 2022; Rawlinson et al., 2022; Dale et al., 2024; White et al., 2024). Such assemblages certainly assist in understanding handaxe variation in MIS 9, yet due to their inherent ‘secondary’ context they are inappropriate for understanding non-handaxe assemblages, unless there is clear evidence of a difference in artefact condition or the stratigraphy of the finds. The lack of recent excavations and the predominance of secondary-context sites has meant that there has been little new evidence that can help answer questions regarding the non-handaxe signature in MIS 9.

This paper aims to answer the following three questions:

1. What are the legitimate MIS 9 non-handaxe assemblages?
2. Are there any technological or typological differences between MIS 9 handaxe and non-handaxe assemblages, other than presence/absence of handaxes?
3. What does this mean in the wider British/European context?

## Material and Methods

We conducted a literature search in order to assess the prevalence of non-handaxe assemblages representing MIS 9, using the following sources and references within: McNabb (2007), Mepham (2009), Pettitt and White (2012), Roe (1968a), White (2000), White and Bridgland (2018), Wymer (1968, 1985, 1999). Additionally, new fieldwork in East Anglia, as part of the Breckland Palaeolithic Project (BPP), led to the excavation of a new potential non-handaxe assemblage at the site of Redhill (Davis et al., 2024).

For an assemblage to be classified as ‘non-handaxe’, it was required that the site, or archaeological context, yielded no evidence of handaxes or their manufacture, such as soft hammer flakes. McNabb (2007) has previously suggested that a minimum of 500 artefacts, ideally 1000, should be required for a site to be classed as a non-handaxe assemblage based on negative evidence of this sort. Whilst such sample sizes would be desirable, in the absence of larger excavated assemblages, it is still important to give serious consideration to smaller assemblages.

Fourteen sites were identified that have potentially yielded non-handaxe assemblages from MIS 10/9 (Table 2). Of these, five sites have not been included in the formal analysis. Both Remenham and Rainbow Bar were discounted based on the mixture of material evident from personal observation of museum collections and previous literature (Draper, 1951; Wymer, 1968; Hack, 1998, 1999, 2000, 2004, 2005; McNabb, 2007). Study of Southacre and Twydall remains outstanding due to lack of access to material and could form the basis of future research, but there is little evidence from the literature that these would differ from the other sites dismissed by this study (discussed below). Furthermore, artefacts from Palmer’s (1975) excavations at Purfleet were not available for study, limiting analysis, and therefore previous literature has been relied upon for comparison (Palmer, 1975; Schreve et al., 2002; Bridgland et al., 2013).

**Table 2:** Sites claimed to contain non-handaxe components in MIS 9

Site	Reason for association with the Clactonian	Reference to ‘Clactonian’	Amount of material examined	Acceptance of non-handaxe status	
				Y/N	Reason
Globe Pit, Little Thurrock	-Lack of handaxes and handaxe manufacture	King and Oakley (1936), Wymer (1957)	565	Yes	-Assemblage shows no signs of handaxe manufacture
Cuxton	- Lack of handaxes and handaxe manufacture in a distinct layer preceding the Acheulean	Cruse et al. (1987)	125 (Cruse 1–6) compared to 165 (Cruse 7 +) and 488 (Tester)	Yes	-Cruse’s layers 1–6 show no signs of handaxe manufacture -Distinct from layers 7 +
Purfleet (Beds 1–3)	-Lack of handaxes and handaxe manufacture in a distinct layer preceding the Acheulean	Palmer (1975); Wymer (1985)	A few examples examined Actual assemblage ~ 100	Yes	-Whilst analysis was limited, nothing contradicted previous work and the site is well recorded by Schreve et al. (2002) and Bridgland et al. (2013) -Equivalent to Globe Pit
Redhill (Basel Gravels)	-Excavated core and flake assemblage	N/A	102	Yes	-Lack of handaxe and handaxe manufacture in basal gravels -Handaxes in historic collections more rolled
Groveland's Pit	- ‘Clactonian artefacts’ including chopper cores and retouched flakes (sometimes referred to as Mousterian) -Claims of distinct condition from handaxes	Barnes et al. (1929), Roe (1981), Wymer (1988)	209	No	-There is no clear distinction between core and flake working and handaxes, and no evidence of a separate assemblage -Flake tools more advanced than other non-handaxe sites
Baker’s Farm	-Small number of artefacts at base of section - Claims of ‘Earliest Clactonian’ and compared to Swanscombe	Breuil (1932), Lacaille (1940)	313	No	-No proven separation from handaxe manufacture -No distinction in condition
Stoke Newington	-Large number of cores and flakes -Chopper cores, denticulates and notches -Clactonian III—advanced ‘Mousterian character’	Warren (1912, 1942)	544	No	-No evidence of separation from handaxe manufacture -No distinction in condition -Flake tools more advanced than other non-handaxe sites
Remenham	-Large core and flake assemblage with minimal evidence of handaxe manufacture	Wymer (1968)	N/A	No	-Two handaxes found alongside assemblage -Mixture with later prehistoric material
Twydall	-Link to MIS 9 Group I sites -Large core and flake collection - ‘Typologically Clactonian artefacts’	Cook and Killick (1924), Beresford (2018)	N/A	?No	-No evidence for a clear separation -Based on Clactonian typologies rather than genuine separation
Biddenham	-Large core and flake assemblage compared to Warren’s collections - ‘Clactonian tortoise and disc cores’ - Basal layers potential source of cores and flakes	Knowles (1953), Harding et al. (1991)	604	No	-No evidence of separation from handaxe manufacture -No distinction in condition
Kempston	-Similarity to Biddenham	N/A	165	No	-No evidence of separation from handaxe manufacture
Barnham Heath	-Mixed site with large core and flake component - ‘Clactonian cores’	Roe (1981)	376	No	-No evidence of separation from handaxe manufacture -No distinction in condition between handaxes and an unprepared core and flake assemblage
Southacre	-Flakes of Clactonian type -Chopper cores and large flakes	Sainty (1935)	N/A	?No	-Based on typological grounds -No clear evidence of separation from handaxe manufacture
Rainbow Bar	-Large core and flake assemblage with no evidence of handaxe manufacture	Draper (1951)	28 (Not formally analysed due to undated and highly mixed nature)	No	-Heavily mixed, including later prehistoric material -Little evidence for dating

Assemblages from the nine remaining sites in this study were recorded in detail, including metric data, condition and technological observations (SOM2; Ashton & McNabb, 1996a, b; Ashton et al., 1998). Key to identifying non-handaxe assemblages was whether they were stratigraphically distinct from any handaxe component, and, if not, whether there was a difference in artefact condition that enabled separation from the handaxe component. Comparisons were then made with a wider corpus of 14 better-dated MIS 9 handaxe assemblages from across England including from the Thames and Solent river systems and Eastern England (Rawlinson, 2021). An examination of the flake tools from MIS 9 contexts has previously been published (Rawlinson et al., 2022), but data from that study is used in the following to further explore potential differences between non-handaxe and handaxe assemblages.

## Results

### Which Are the Legitimate Non-handaxe Assemblages?

Only the three assemblages previously discussed by White (2000), Globe Pit (Little Thurrock), Cuxton (1–6) and Purfleet (Little Thurrock member), and the newly excavated assemblage at Redhill, could be verified as discrete non-handaxe assemblages.

**Fig. 2:**
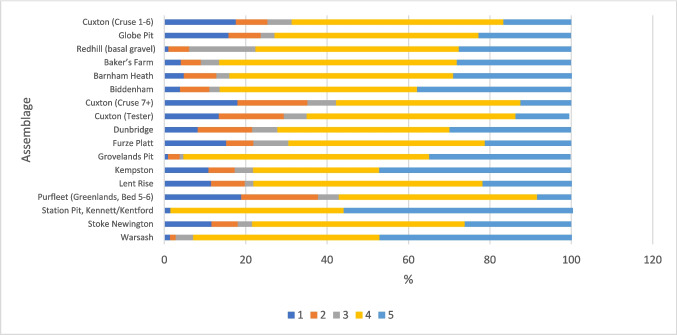
Proportion of flake types across the assemblages studied (1 = early stage working/cortical to 5 = late stage working; see SOM2)

The six other sites did not contain discrete core and flake assemblages based on stratigraphy or condition (Tables 2, 3, and 4), and have been argued to contain Clactonian elements on dubious techno-typological grounds (see Table 1). The common consensus that such ‘positive identifiers’ have little validity (McNabb, 1992; White, 2000; Cole, 2011; Fluck, 2011) is followed here, although a technological comparison is presented in the following. The non-handaxe assemblages contain all stages of working, indicated through flake types (Fig. 2; the low proportion of flake type 1 at Redhill is discussed in the following). This is also true of most handaxe assemblages, but Station Pit and Groveland’s Pit show a lack of early stage working, which could show sites where only the later stages of handaxe manufacture took place, but this most likely reflects collection bias (Rawlinson, 2021).

**Table 3:** Number and condition of whole hard hammer and soft hammer flakes, and flake and butt type of hard hammer flakes

Sites	*n*	# soft hammer	Condition					
			Hard hammer			Soft hammer		
			Fresh	S. Rolled	Rolled	Fresh	S Rolled	Rolled
Cuxton (Cruse 1–6)	102	0	70.9	26.4	2.7	-	-	-
Globe Pit	493	0	16.5	80.9	2.5	-	-	-
Redhill (basal gravel)	42	0	40.48	47.62	11.9			
Baker’s Farm	221	19	10	75.6	14.4	31.6	63.2	5.3
Barnham Heath	251	17	4.6	90	5.4	29.4	64.7	5.9
Biddenham	433	36	2.7	87.2	10.1	16.2	83.8	-
Cuxton (Cruse 7 +)	128	14	50.7	40.4	8.9	62.5	31.3	6.3
Cuxton (Tester)	357	7	22.1	67.2	10.7	42.9	57.1	-
Dunbridge	97	7	1.8	86.4	11.8	28.6	71.4	-
Furze Platt	269	26	14.8	69.4	15.8	50	46.2	3.8
Groveland's Pit	101	6	6	81.9	12.1	-	100	-
Kempston	110	3	1	69.6	29.4	33.3	66.6	-
Lent Rise	96	5	4.3	68.7	27	60	40	-
Purfleet (Greenlands, Bed 5–6)	44	15	46.3	53.7	-	80	20	-
Station Pit, Kennett/Kentford	135	13	9	54.5	35.2	41.7	50	8.3
Stoke Newington	431	7	12	78.7	9.3	57.1	42.9	-
Warsash	72	14	11	69.9	19.2	35.7	64.3	-

**Table 4:** Technological summary of cores from MIS 9

Site	*n*	Type of core					Average number of core episodes	Average number of removals
		MPC	Chopper	Discoidal	Fragment	Misc		
Non-handaxe assemblages								
Cuxton (Cruse 1–6)	4	100	-	-	-	-	2.25	7.25
Globe Pit	10	90	-	-	-	10	2.10	4.30
Redhill (basal gravel)	2	50	-	-	-	50	1.50	4.00
Handaxe assemblages								
Baker’s Farm	3	100	-	-	-	-	2.33	6.33
Barnham Heath	32	75	9.38	-	-	15.23	2.47	5.75
Biddenham	13	53.85	-	30.77	-	15.38	2.46	6.92
Cuxton (Cruse 7 +)	3	66.6	-	-	-	33.3	1.67	3.33
Cuxton (Tester)	23	65.22	30.43	-	4.34	-	1.91	4.30
Dunbridge	14	85.71	7.14	-	-	7.14	2.79	6.79
Furze Platt	2	100	-	-	-	-	3	6
Groveland's Pit	28	85.71	7.14	-	3.57	3.57	2.71	7.21
Kempston	5	80	20	-	-	-	2.2	7
Lent Rise	1	100	-	-	-	-	1	4
Purfleet (Greenlands, Beds 5–6)	4	100	-	-	-	-	3.5	7
Station Pit, Kennett/Kentford	5	100	-	-	-	-	2.2	7.4
Stoke Newington	13	76.92	7.69	-	15.38	-	1.85	4.46
Warsash	8	75	12.5	12.5	-	-	2	6.5

**Fig. 3:**
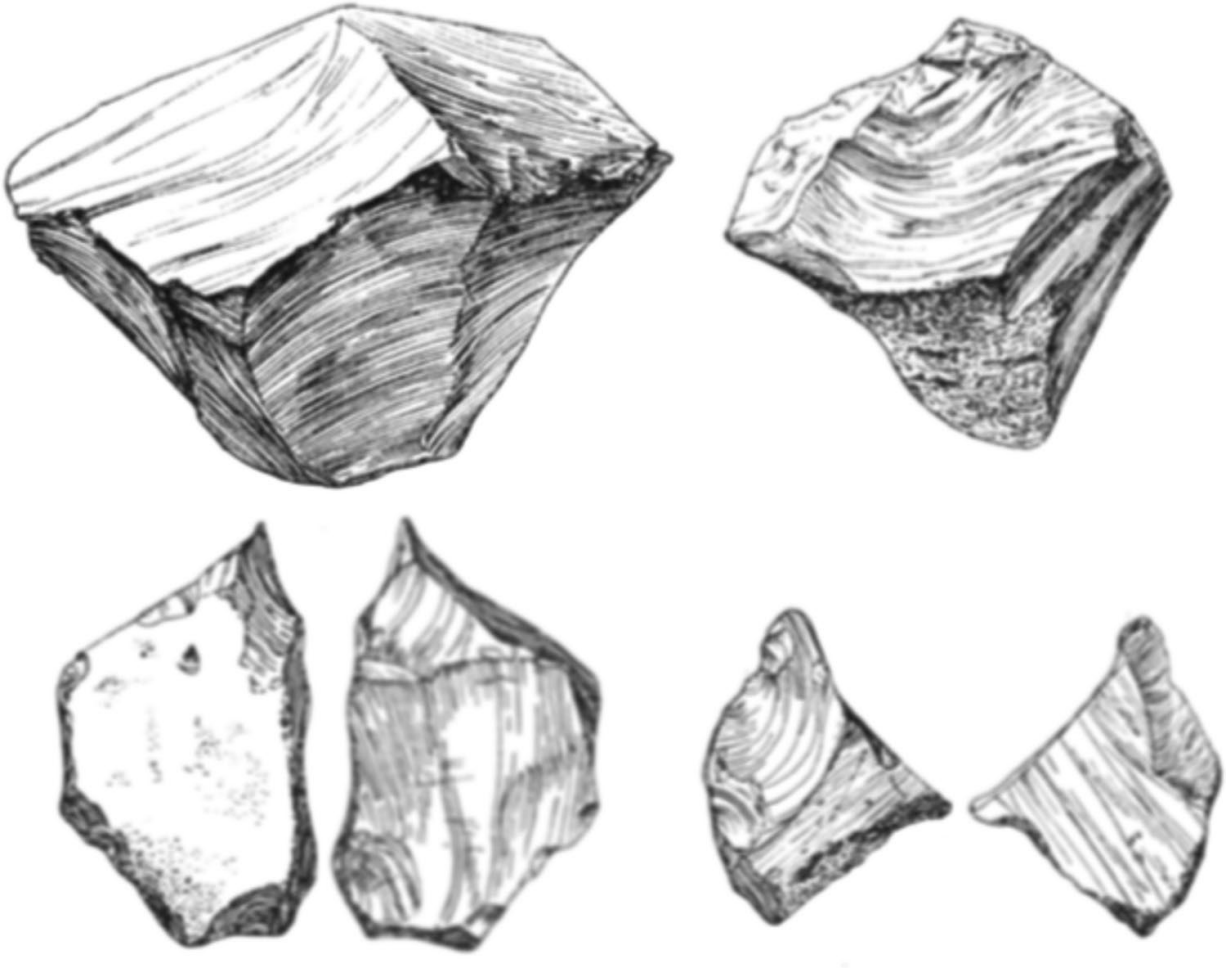
Examples of cores and flakes from Wymer’s excavations at Little Thurrock (Wymer Archive, British Museum)

Globe Pit (Little Thurrock) represents the most convincing evidence for a non-handaxe site, with over 1000 artefacts (Fig. 3). No evidence for handaxe manufacture was found in the 565 artefacts (flakes, flake tools and cores) examined in this study. The site’s lateral equivalent at Purfleet (Little Thurrock member) has yielded a smaller assemblage (~ 100), but has been subject to multiple modern excavations over a ~ 500-m stretch of the MIS 9 Thames deposits (Palmer, 1975; Schreve et al., 2002; Bridgland et al., 2013; White & Bridgland, 2018). Palmer (1975) originally mentioned a Clactonian element within a Middle Acheulean assemblage. Unlike at other handaxe sites, re-evaluation of Purfleet has shown that there is a distinct non-handaxe assemblage in the basal gravels prior to the appearance of the handaxe industry (Schreve et al., 2002; Bridgland et al., 2013).

The site at Cuxton has been argued to contain the same tripartite sequence as Purfleet (White & Bridgland, 2018). No separation by either condition or stratigraphy could be made in the assemblage from Tester’s (1965) excavation, but the Cruse et al. (1987) material from layers 1–6 with 125 artefacts was in a fresher condition with no soft hammer flakes, unlike the assemblage of 165 artefacts with handaxes from layer 7 +. It is likely that Tester’s excavation (488 artefacts) only exploited these higher layers.

**Fig. 4:**
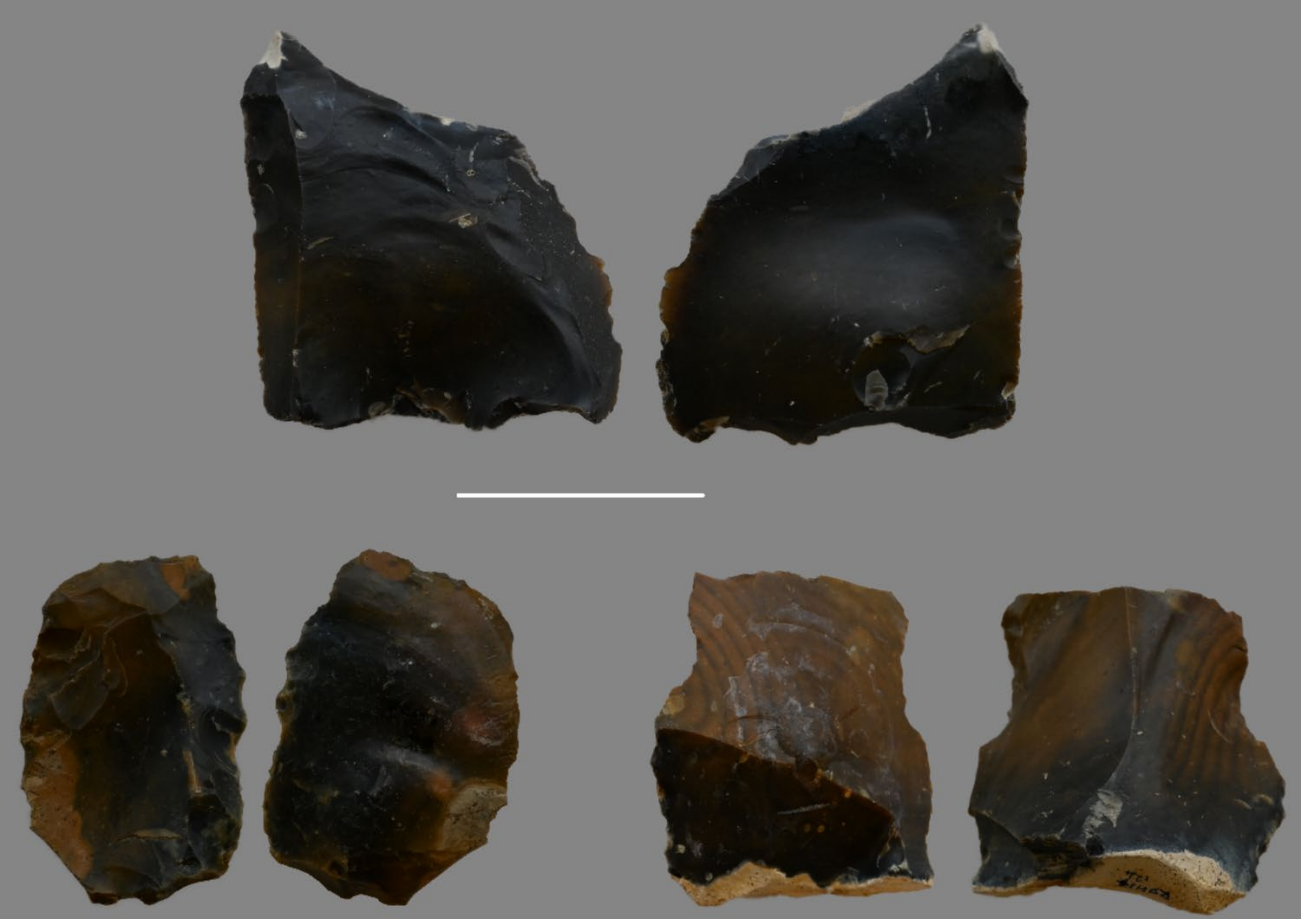
Flake (top), scraper (bottom left) and double notch from BPP excavations at Redhill (scale 5 cm)

The lack of non-handaxe assemblages outside of the Thames has been a major question regarding evidence for the signature during MIS 10/9 (White & Bridgland, 2018). However, Davis et al. (2024) have identified a core and flake assemblage, lacking evidence for handaxe manufacture, in the coarse basal deposits at Redhill, Thetford, in the valley of the River Little Ouse (Fig. 4). The potential for a Clactonian assemblage at this site was not mentioned by Roe (1981) or Wymer (1985). The site has been dated to MIS 10-9-8, and the basal deposits to late MIS 10 or early MIS 9 (Davis et al., 2024), linking it chronologically to the non-handaxe assemblages in the Thames. The archaeology is in a fresh condition, with historically collected handaxes from the site being more rolled. Redhill is the only potential non-handaxe site of MIS 9 age outside of the Thames and would represent a significant advancement in our knowledge of this period.

Whilst these four sites contain evidence for non-handaxe assemblages during late MIS 10 / early MIS 9, other sites that have previously been suggested as containing ‘non-handaxe components’ or ‘Clactonian affinities’ do not stand up to scrutiny. Whilst there are differences between the condition of hard hammer flakes and soft hammer flakes at a number of these sites (Table 3), with soft hammer flakes showing less abrasion, this is likely due to their smaller size and resulting taphonomy with more rolled examples having gone unnoticed, been winnowed away or being undiagnostic. The handaxes themselves are more closely aligned with the more abraded and more varied hard-hammer flakes (Dale, 2022). When considered alongside the technology (discussed in the following), the evidence for any distinct non-handaxe assemblage is weak.

In the Thames Valley, Groveland’s Pit, Baker’s Farm and Stoke Newington were argued to have Clactonian elements due to their high proportions of cores, flakes and flake tools (Breuil, 1932; Lacaille, 1940; Roe, 1981; Wymer, 1968, 1985), but there is no evidence of separate assemblages at any of these sites, either from contextual records or from distinctions in condition.

One site which was not evaluated in full is Twydall, Kent. A recent study by Beresford (2018) has linked the site to MIS 9 after work reaffirmed Roe’s (1968b) Group I classification of the handaxes, akin to other MIS 9 sites (White & Bridgland, 2018; Dale, 2022). Yet, as cores and flakes represent over 80% of the material, there have also been suggestions of a Clactonian industry (Roe, 1981; Beresford, 2018). Despite this, there has been no demonstration of a distinct non-handaxe layer with handaxes and cleavers found amongst the material. Unless a stratigraphic separation can be demonstrated, the site cannot be confirmed to represent a non-handaxe signature.

Beyond the Thames, the sites of Biddenham, Kempston, Southacre and Barnham Heath have all been previously suggested to have yielded Clactonian components due to the large numbers of cores, flakes and flake tools, including chopper cores, notches and denticulates, all previously regarded as diagnostic of the Clactonian (Sainty, 1935; Knowles, 1953; Roe, 1968a; Wymer, 1985). There is no evidence of separate non-handaxe assemblages and currently all the evidence from these sites is consistent with that from handaxe sites across the British Lower Palaeolithic. However, at Biddenham, the geological sequence contains a basal layer that has been suggested to be the source of a non-handaxe assemblage (Harding et al., 1991). Whilst nothing has been found to contradict this, the number of excavated and well provenanced artefacts is very low. The soft hammer flakes are less abraded, but as explained above, this is in line with other sites and is most likely taphonomic. It would be possible to test the potential of the suggested sequence at Biddenham through excavation at the SSSI Deep Spinney Pit. Recent fieldwork at Barnham Heath has not found convincing evidence to suggest there is a distinct core and flake assemblage within the material from here (Davis et al., 2024). Therefore, whilst there is potential at a number of sites, previous references to ‘Clactonian working’ are probably due to outdated understanding of the Clactonian based on an abundance of cores and flakes with diagnostic features such as chopper cores and notches, rather than a genuine separation from evidence of handaxe manufacture.

There have been few claims of Clactonian / non-handaxe assemblages in the Solent due to the dominance of handaxes in the record (Hosfield, 1999, 2001; Wymer, 1999). Roe (2001) argued that individual industries were likely to be mixed in the Solent. This has led to a poorer understanding of the stratigraphy and age of many of the sites. Recent work has tried to rectify this but there have been no claims of non-handaxe assemblages (Westaway et al., 2006; Davis et al., 2016, 2021; Hatch et al., 2017). Rainbow Bar is the only site suggested to be linked to the Clactonian (Roe, 2001). The undated and highly mixed nature of this site makes it a poor candidate to examine, with reports of handaxes (Hack, 2000, 2004), Levallois (Draper, 1951), later prehistoric material and naturally flaked flint (McNabb, 2007).

### Are There Any Technological or Typological Differences Between Handaxe and Non-handaxe Assemblages, Other Than Presence/Absence of Handaxes?

The only major difference between flakes from non-handaxe contexts and those from handaxe contexts is the presence/absence of soft hammer working (Table 3; cf. Bridgland et al., 2013 for Purfleet). Many of the differences between the technology and typology of the handaxe and non-handaxe assemblages can be explained by collection bias; the excavated or better collected assemblages include the full range from simpler flakes to those with clearer signs of working, as well as smaller artefacts that could have been missed in older collections. The two different assemblages from Cruse’s et al. (1987) excavation at Cuxton show little technological distinction apart from the absence/presence of soft hammer flakes, demonstrating how this could be overlooked if the assemblages were to become mixed.

**Fig. 5:**
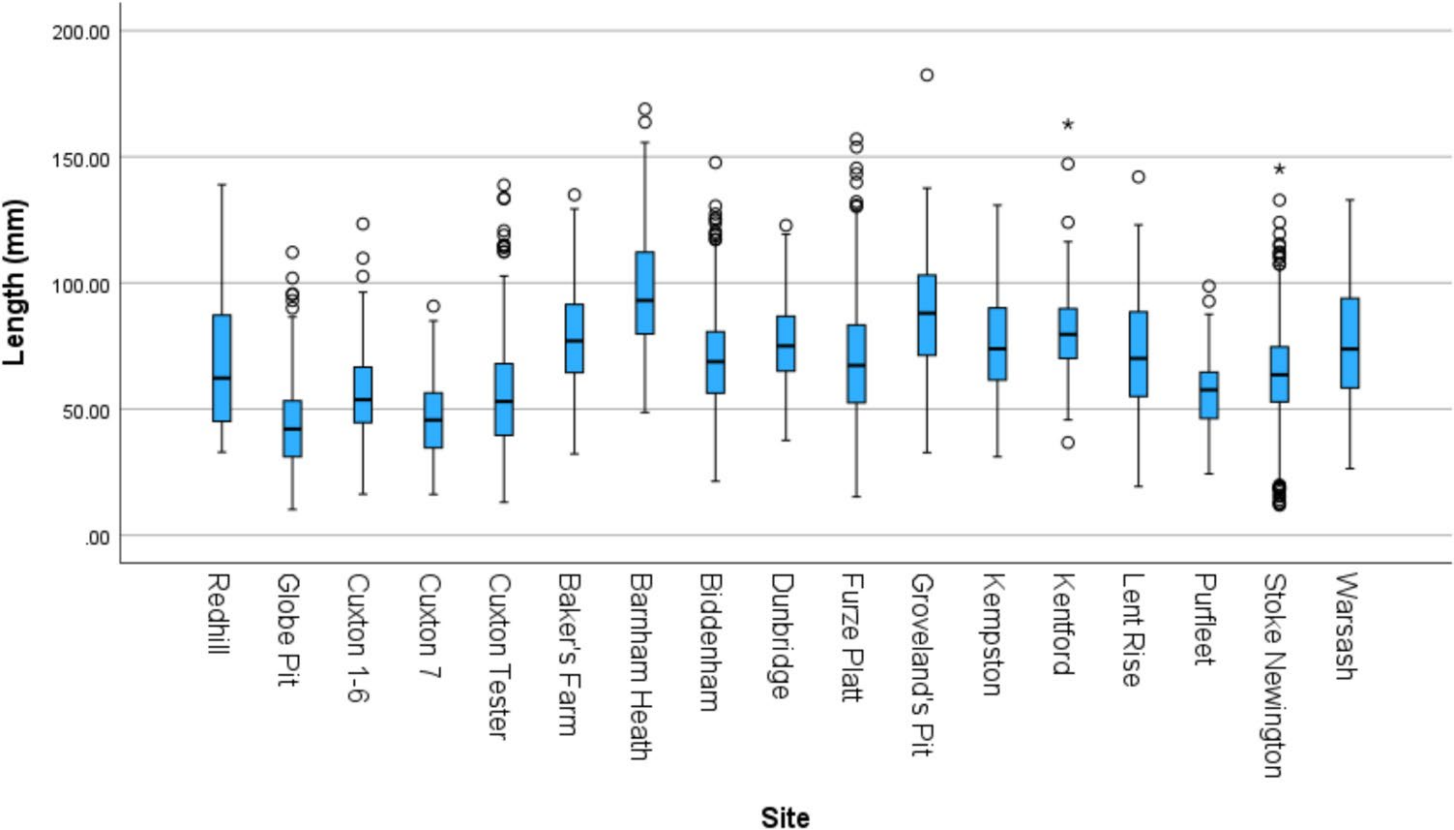
Boxplot of flake length of MIS 9 sites (Non-handaxe assemblages on the left)

**Fig. 6:**
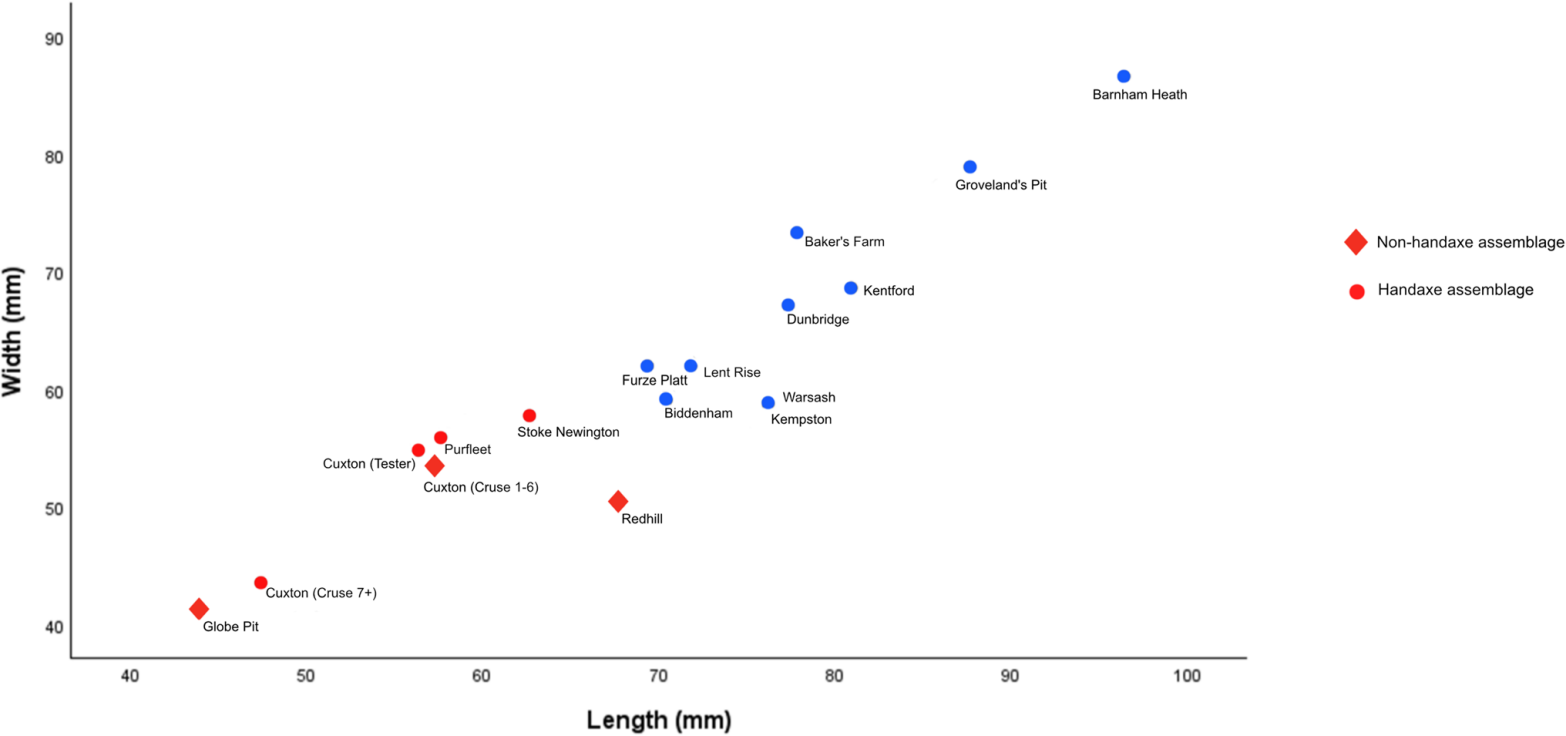
Length and width of flakes from MIS 9 contexts (red, excavated/carefully collected; blue, collected/secondary context)

Differences in flake size are most likely due to the presence/absence of soft-hammer flakes; soft-hammer flakes were therefore removed from the flake-size comparison (Table 5). On face value, Fig. 5 demonstrates that average flake length is often smaller in non-handaxe assemblages. However, the size of flakes appears to be more influenced by the method of collection rather than assemblage type. Figure 6 shows a clear trend of assemblages which are excavated or carefully collected being smaller on average than the historic collections from secondary-context sites. As would be expected, the excavated assemblages contain smaller flakes, followed by those that were carefully collected (e.g. Stoke Newington). Whilst Globe Pit has the smallest flakes on average, the next closest sites are the handaxe layers in the Cruse excavations at Cuxton.

**Table 5:** Average measurements of hard hammer flakes from MIS 9 sites with standard deviations

Site	*n*	Length (mm)		Width(mm)		Thickness		Elongation (W/L)	
		Mean	Std. deviation	Mean	Std. deviation	Mean	Std. deviation	Mean	Std. deviation
Non-handaxe assemblages									
Cuxton 1–6	102	57.26	20.121	53.47	21.259	17.44	8.268	0.97	0.342
Globe Pit	493	43.93	16.146	41.29	15.137	13.90	6.298	0.99	0.347
Redhill	42	67.69	28.350	50.40	19.549	17.99	9.543	0.79	0.244
Handaxe assemblages									
Baker’s Farm	221	77.83	19.083	73.32	21.106	20.04	9.250	0.98	0.320
Barnham Heath	251	96.40	24.481	86.63	25.198	27.18	10.811	0.93	0.292
Biddenham	433	70.45	19.852	59.17	19.283	17.01	7.437	0.88	0.293
Cuxton (7 +)	128	47.41	16.546	43.60	17.693	13.37	6.738	0.95	0.336
Cuxton (Tester)	357	56.34	21.821	54.84	23.097	17.00	9.132	1.01	0.330
Dunbridge	97	77.34	18.688	67.14	16.951	23.38	8.264	0.90	0.260
Furze Platt	269	69.36	26.564	62.01	23.200	18.88	9.557	0.95	0.328
Groveland’s Pit	101	87.64	23.031	78.93	25.306	26.00	8.668	0.93	0.293
Kempston	110	76.19	19.940	58.90	18.668	18.85	8.151	0.82	0.306
Kentford (Station Pit)	135	80.93	18.665	68.64	19.650	21.17	7.586	0.88	0.272
Lent Rise	96	71.82	22.331	62.00	20.742	19.50	8.439	0.91	0.339
Purfleet (Greenlands, Beds 5–6)	44	57.63	16.819	55.97	20.013	20.12	9.692	1.00	0.305
Stoke Newington	431	62.66	21.453	57.81	20.594	17.46	8.343	0.97	0.319
Warsash	72	76.20	24.428	58.79	19.854	17.94	11.224	0.84	0.395

When taking the two excavated assemblages from Cruse’s excavation at Cuxton, the non-handaxe assemblage shows larger flakes, but this is still not a major distinction. This is further backed up by an ANOVA test, a TukeyHSD post hoc analysis and a cluster analysis, which showed that the majority of the assemblages were significantly different from each other (SOM3). Similarities were found between assemblages that had been excavated (including Cruse’s two distinct Cuxton assemblages) as opposed to collected assemblages. There was no indication of a difference based on the classification of the site as non-handaxe or handaxe. Variation is more likely to be influenced by natural differences in site formation, raw material or collection history. Larger well-excavated assemblages from primary-context sites would be better to identify any meaningful differences between sites with and without handaxes. This would optimally involve a wider analysis incorporating the MIS 11 Clactonian data, although previous attempts have shown no distinguishable differences (McNabb, 2007; Cole, 2011; Fluck, 2011).

**Fig. 7:**
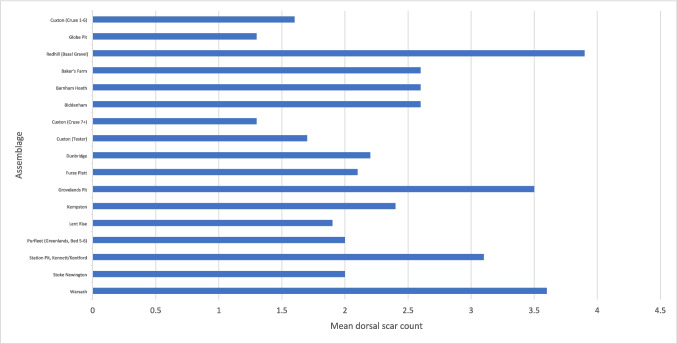
Mean dorsal scar count by assemblage

**Fig. 8:**
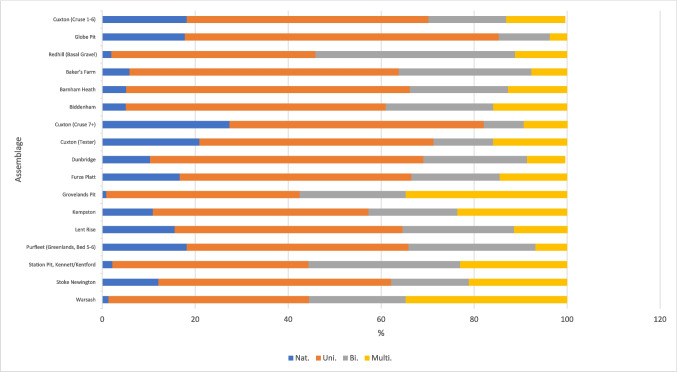
Proportion of dorsal scar pattern by assemblage

**Fig. 9:**
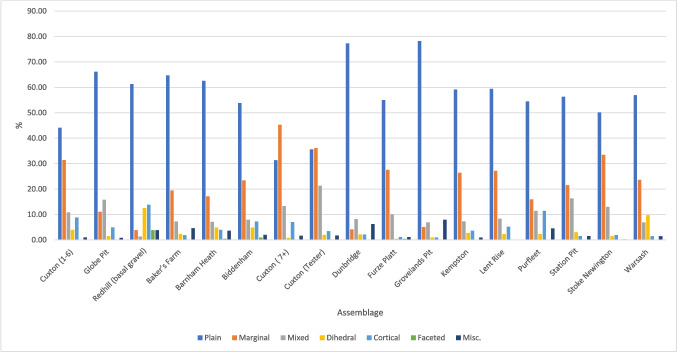
Proportions of butt type by assemblage

When comparing hard-hammer flakes from both assemblage types, there are differences in proportion of flake type, dorsal-scar pattern, average dorsal-scar count and butt type, but these do not always show simpler working in non-handaxe contexts. Flakes from handaxe assemblages often have a higher average dorsal-scar count and higher proportions of flakes with more complex dorsal-scar patterns that might result from the earlier stages of handaxe manufacture with a hard hammer, rather than from core working (Figs. 7 and 8). However, more complex dorsal-scar patterns with higher dorsal-scar counts are not absent from non-handaxe assemblages and so cannot form a diagnostic feature of an assemblage. There are no diagnostic features in butt working with plain and marginal butts being the majority (Fig. 9). There are signs of more intensive working at both the non-handaxe sites (Redhill) and handaxes sites (Biddenham and Warsash) with evidence of dihedral butts and occasional examples of faceting.

The Redhill assemblage shows more intensive working than those from the other non-handaxe sites, which could be related to raw material at the site. The basal gravel is coarse, including nodular flint, which may have enabled more heavily reduced cores, leading to greater proportion of non-cortical flakes. Redhill has similarities to handaxe sites that lack the earlier stages of working, such as at Groveland's Pit and Station Pit (Kentford), which may in turn lack earlier stages of working due to the collection bias discussed above. At Redhill, this could be due to Davis et al.’s (2024) use of Lubinski et al.’s (2014) strict method of distinguishing flakes from geofacts, which cautiously precludes early stages of manufacture. The heavily reduced nature of the Redhill material could explain the presence of small numbers of flakes classified as having faceted butts. Whilst these show butts with scars from previous working, they do not appear to show preparation and are simply flakes from well-exploited cores.

The small number of cores from all sites is probably due to collection bias, with cores only retained if they had an obvious or interesting form at most sites, although cores also are proportionally low in excavated assemblages such as Cuxton and Redhill. This makes drawing conclusions from the metrical data more difficult, but a similar pattern emerges with most excavated sites showing smaller cores on average (Table 6). The larger size of the cores from the non-handaxe layers of Cuxton could be due to raw material, as they also show a higher level of removals than other sites, precluding the idea that these were larger due to lack of working. Alternatively, this could simplify the result of a small sample.

**Table 6:** Average measurements of cores from MIS 9 sites

Sites	*n*	Length (mm)	Width (mm)	Thickness (mm)	Elongation (W/L)	Flattening (Th/W)
Non-handaxe assemblages						
Cuxton (Cruse 1–6)	4	104.7	95.6	55.1	1.017	0.643
Globe Pit	10	72.6	52.1	33.2	0.751	0.643
Redhill (basal gravel)	2	70.85	51.55	32.6	0.73	0.641
Handaxe assemblages						
Baker’s Farm	3	97.3	72.8	36.1	0.768	0.494
Barnham Heath	32	121.5	108.0	57.7	0.948	0.582
Biddenham	13	94.3	78.6	31.7	0.867	0.413
Cuxton (Cruse 7 +)	3	78.1	57.2	48.8	0.725	0.829
Cuxton (Tester)	23	100.7	73.5	48.0	0.755	0.682
Dunbridge	14	105.7	95.7	52.9	0.943	0.579
Furze Platt	2	123.8	91.1	57.6	0.737	0.636
Groveland's Pit	28	112.6	100.4	56.7	0.931	0.597
Kempston	5	86.1	84.4	51.0	0.985	0.589
Purfleet (Greenlands, Bed 5–6)	4	123.4	96.6	70.2	0.830	0.712
Station Pit, Kennett/Kentford	5	95.5	78.0	36.9	0.826	0.500
Stoke Newington	13	92.1	68.7	43.3	0.846	0.645
Warsash	8	95.0	81.7	54.1	0.850	0.709

**Fig. 10:**
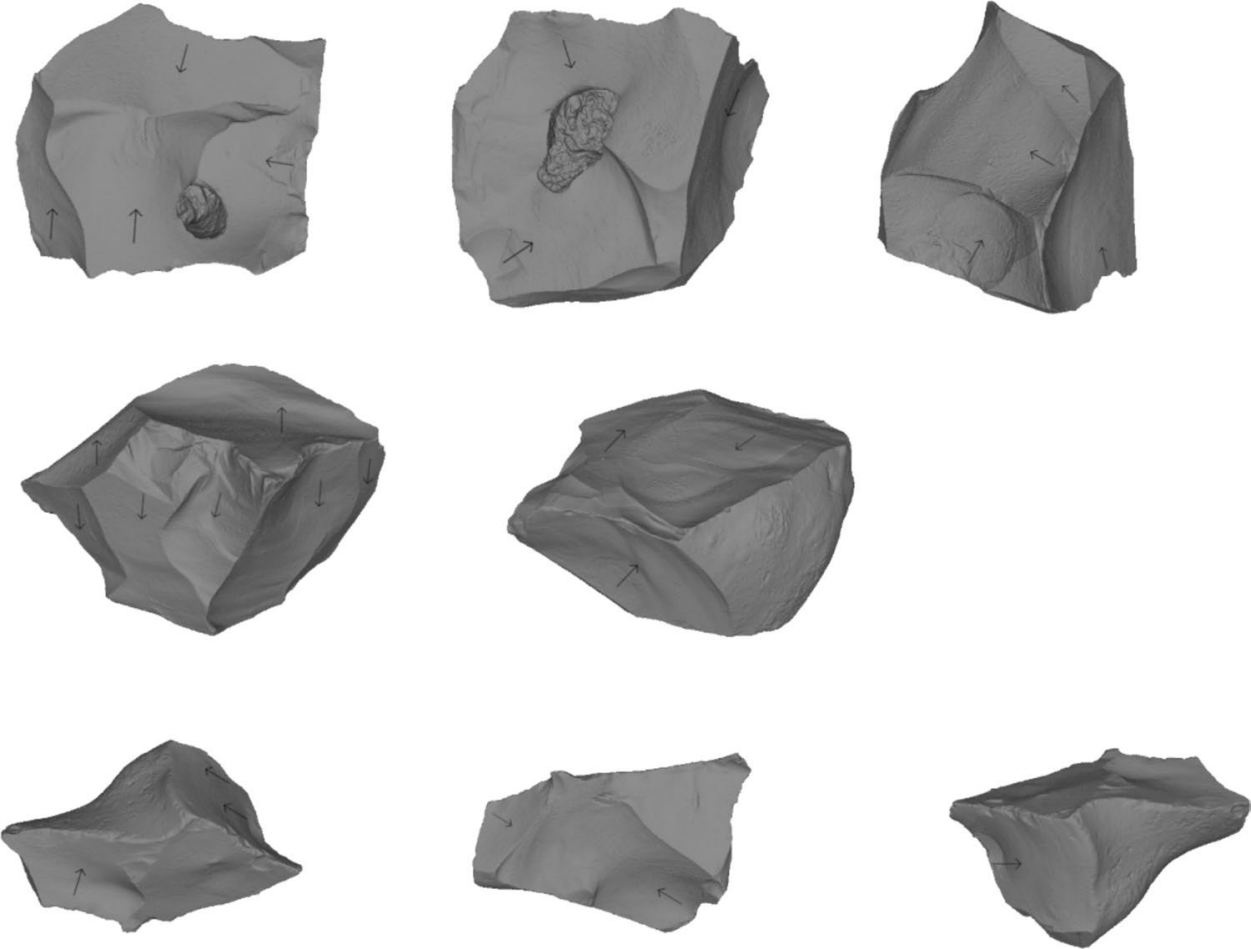
Cores from MIS 9 non-handaxe assemblages (top, Cuxton 1–6 (length 63.3 mm); middle, Globe Pit (length 86.5 mm); bottom, Redhill (length 69.2 mm))

Core working associated with SPC/Levallois has not been included here, although this was considered in detail by White et al. (2024). The cores from Cuxton (1–6), Globe Pit (Little Thurrock), Redhill and Purfleet-Little Thurrock Member (Bridgland et al., 2013) are Migrating Platform Cores (MPCs), or simple cores made on fragments, showing moderate levels of exploitation (Table 4; Fig. 10). These cores show the use of parallel and alternative knapping episodes, as well as isolated removals, typical of the Lower Palaeolithic.

Assemblages discussed as having ‘Clactonian elements’, such as from Stoke Newington and Groveland's Pit, contain chopper cores, but these were also found in the excavated handaxe context at Cuxton. The absence of supposed chopper cores from the excavated non-handaxe assemblages undermines their use as a marker for that assemblage type, particularly as they are known from multiple handaxe contexts (Table 4; White et al., 2024). The example from Globe Pit (Fig. 10) shows alternative working on one end of the core, as is commonly seen in chopper cores. However, additional removals from distinct platforms indicate that this is just one alternative knapping episode on a MPC. The work of Ashton et al. (1992) demonstrated that chopper cores could be explained as a coincidental outcome of intensive alternative knapping. Despite being previously linked to Clactonian working, discoidal cores are also not known from the MIS 9 non-handaxe sites, although they are present at Biddenham and Warsash (Table 4). Again this shows the occurrence of different core shapes across the Lower Palaeolithic (White et al., 2024). The average number of core episodes and number of removals show little distinction in core working between non-handaxe and handaxe assemblages.

In sum, the non-handaxe assemblages show few biases in stages of working but there is some evidence for predominantly short and simple chains of removal, although these are not clearly distinct from wider variation within MIS 9.

**Fig. 11:**
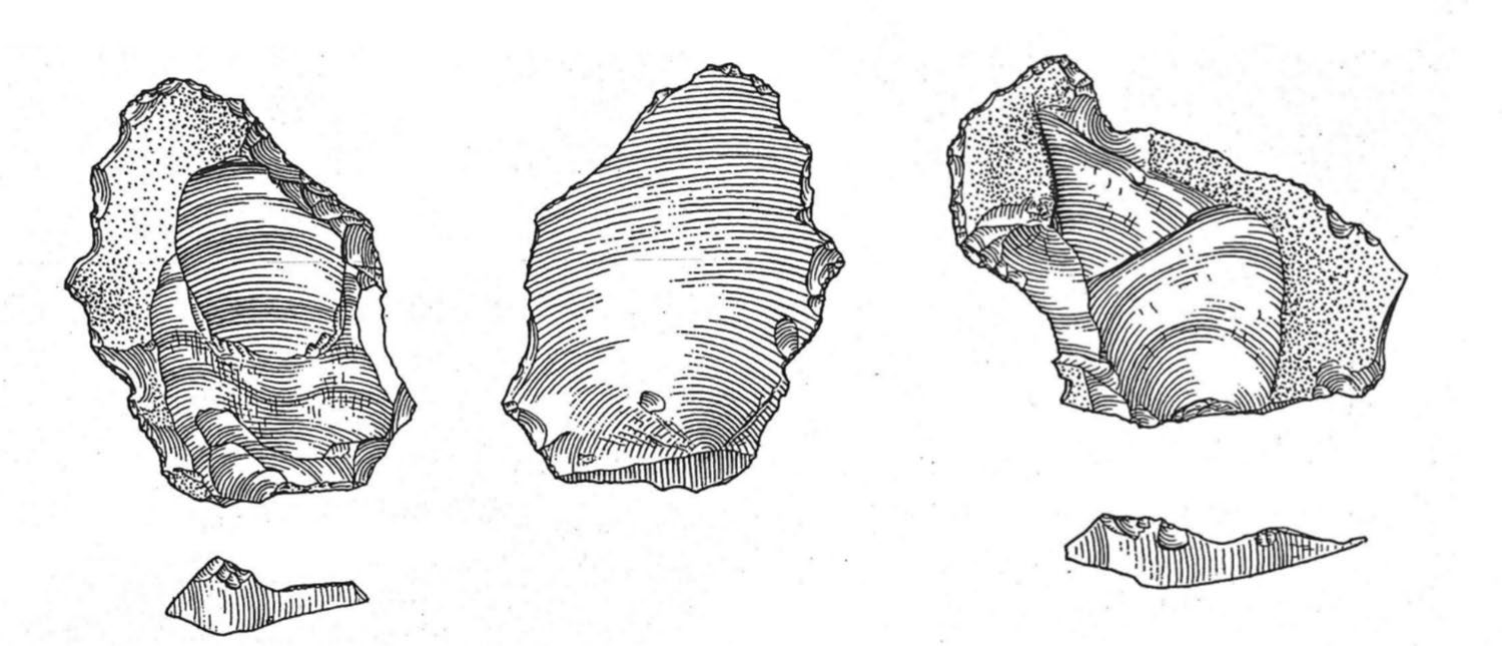
Two simply retouched flake tools from non-handaxe layers at Cuxton (Cruse archive, British Museum)

**Fig. 12:**
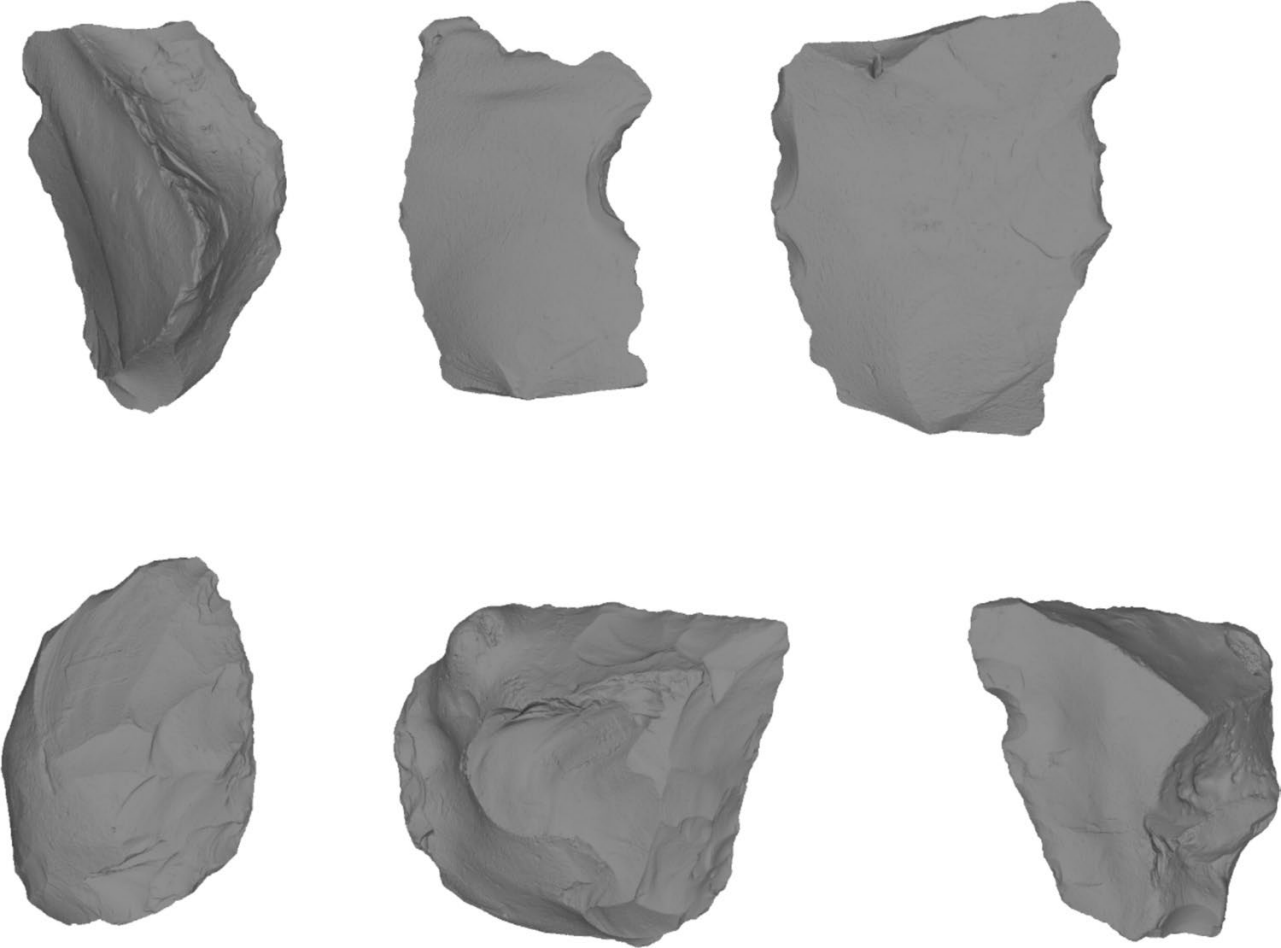
Examples of flake tools from Redhill (top right to left; minimally invasive side scraper (length 83.5 mm), notch (length 81.9 mm), denticulate (length 74.5 mm) and Stoke Newington (bottom, two invasively worked scrapers (length 79.8.6 mm and 63.7 mm) and a simple notch (Length 69.1 mm)

Rawlinson et al. (2022) demonstrated that few characteristics separate flake tools associated with these core-and-flake assemblages from those associated with handaxes. Both assemblage types are dominated by scrapers with small proportions of other flake tool types such as denticulates and notches. Flake tools from the non-handaxe sites include types previously suggested to be diagnostic of the Clactonian, such as notches, denticulates and other simple flake tools. However, these are also common across handaxe sites in MIS 9. The only potential distinction is the shorter retouched edges and the comparative rarity of invasively retouched flake tools associated with core and flake assemblages (Rawlinson et al., 2022; Fig. 11). The newly excavated assemblage from Redhill yielded 15 flake tools, which conform to these observations, with scrapers (33.3%) being the most common form of flake tool, together with notches (21%), denticulates (26.7%) and two flakes which show a combination of retouch types, but no invasively retouched tools (Davis et al., 2024; Fig. 12).

Warren (1942) argued that the Stoke Newington assemblage contained evidence of an advanced Clactonian (Clactonian III) with well-made flake tools (Fig. 12). The flake tools from Stoke Newington, and the other sites, do not differ in condition to the handaxes and thinning flakes, and have been argued to be characteristic of the Acheulean assemblages (Kelley, 1937; Rawlinson et al., 2022). Flake tools at Stoke Newington, and a number of other handaxe assemblages, are occasionally ‘well made’ or invasively worked, demonstrating an association with handaxe manufacture, rather than with their absence. However, it is important to note that flake tools in Acheulean contexts are usually still simple ad hoc tools similar to those from non-handaxe contexts.

### Summary

Cuxton, Globe Pit (Little Thurrock), Purfleet and Redhill differ from the sites dismissed above, including Stoke Newington, Groveland’s Pit and Baker’s Farm in the Thames, as well as Biddenham, Kempston and Barnham Heath in the east of England (Table 2), which have only previously been referred to as Clactonian based on perceived typological grounds rather than a demonstratable separation based on context or condition (McNabb, 1992, 2007, 2020). Re-analysis concurs that, like the Clactonian of MIS 11, Globe Pit (Little Thurrock), Cuxton and Purfleet all yielded assemblages with no evidence of handaxe manufacture, but otherwise indistinguishable from traditional Acheulean assemblages in terms of core working and flake-tool production. These appear to be contemporaneous at the end of MIS 10 and/or early in MIS 9. Redhill can also be added to the list of potential non-handaxe sites dating from MIS 10/9, and is the first outside the Thames.

## Discussion

McNabb (2007, 2020) has been understandably cautious about the MIS 9 non-handaxe assemblages for a number of reasons. Sample size is a major concern, with Cuxton (~ 120), Purfleet (~ 100) and Redhill (~ 100). For Cuxton, this is also due to the small size of the area from which the non-handaxe assemblage was excavated having the potential to have missed evidence for handaxe manufacture if it was a smaller component of the technology at the site, especially if it was spatially separated from core working. The same concern is applicable to Redhill. The opposite is true at Purfleet, where the ~ 500-m-long excavated face is a sufficiently large area, but the density of artefacts is low. For McNabb (2020), this possibly means that evidence of handaxe manufacture could be harder to detect. Globe Pit (Little Thurrock) has a substantial assemblage (> 1000), but the time-depth of the sediments has been questioned as the assemblage was excavated from channel-margin sediments which may represent a short period of time rather than being evidence of a long tradition (cf. Swanscombe Lower Gravels). McNabb (2007, 2020) does not dispute the non-handaxe characteristic of the MIS 9 sites, but rather their classification as Clactonian and wider significance. Whilst individually these sites all have potential issues, the lack of handaxes in contemporaneous deposits means that this is a pattern worth deeper examination. The potential addition of Redhill in East Anglia only adds to this pattern, although, as this is a newly discovered assemblage, alternative explanations including raw material influence should be considered.

The MIS 11 sites, like those from MIS 9, lack any form of characteristic tool and the only distinction is the lack of handaxe manufacture (Wenban-Smith, 2013; Ashton et al., 2016; McNabb, 2020). It is therefore not possible to relate the two periods together through the material culture. McNabb (2020) has used this lack of a positive distinction to question the cultural importance of Clactonian/non-handaxe assemblages. We cautiously treat the non-handaxe signature during MIS 10/9 as separate, and therefore do not use the label Clactonian, although the possibility of a link is explored in the following.

Nevertheless, in both MIS 11 and MIS 9, it appears that, early in the interglacial, groups of hominins who did not habitually manufacture handaxes were present at an earlier time than those who utilised handaxe manufacture later in the interglacial, as suggested by White and Schreve (2000). Recent environmental and dating work on MIS 11 sites (Ashton et al., 2008, 2016; Candy et al., 2014; Horne et al., 2023; White et al., 2018, 2023) has given a much clearer picture than that available for MIS 9, although the evidence from Cuxton and Purfleet, as well as the stratigraphy from the MIS 9 sites, supports this hypothesis (White & Schreve, 2000; Bridgland et al., 2013; White & Bridgland, 2018). Based on current evidence then, the MIS 10/9 non-handaxe signature suggests the chronological separation required by Ashton et al. (2005), similar to that in MIS 11.

### Britain’s Place in Europe

Debates around the Clactonian have often felt parochial, but it is becoming increasingly clear that non-handaxe assemblages are a part of the wider European Lower Palaeolithic. The rapid technological turnover in Britain has often been interpreted as evidence of the arrival of a new cultural group from continental Europe (White & Schreve, 2000; Ashton et al., 2016; Ashton & Davis, 2021). The issue is one of correlation between Britain and continental Europe, where the division is less clear (Ashton et al., 2016).

Eastern and central Europe are traditionally seen as areas populated by non-handaxe-making groups and possible sources for non-handaxe groups moving into Britain (Collins, 1969; White, 2000). In contrast, France, Spain and Italy are considered Acheulean strongholds, where potential non-handaxe assemblages such as Tayacian or Colombanien sites have been explained by a flexible Acheulean, raw material or site use (Cook et al., 1982; Rolland, 1986; Monnier & Molines, 1993; Monnier, 1996; Palma di Cesnola, 1996; Abbazzi et al., 2000; Ravon et al., 2016a, b).

Ashton et al., (2016; cf. Fluck, 2011) discussed 14 European non-handaxe sites that have yielded over 50 artefacts each and date from the late Middle Pleistocene. The current evidence shows the predominance of non-handaxe sites in central and eastern Europe with occurrences in France, Italy and Spain (Ashton et al., 2016; Davis & Ashton, 2019; Ashton & Davis, 2021). These authors argued that the paucity of good-quality raw material in central Europe (Rocca et al., 2016) could have led to hominin populations that shared wider behavioural characteristics with contemporary groups elsewhere but did not manufacture handaxes. Many sites in the traditional Acheulean area of western Europe have been argued to have raw-material or functional explanations for the lack (or low numbers) of handaxes (Ashton & Davis, 2021) including Terra Amata (de Lumley et al., 2015), Menez-Dregan (Monnier et al., 1996; Ravon et al., 2016a, b, 2022) and Caune de l’Arago (de Lumley & Barsky, 2004; Barsky & de Lumley, 2010; Barsky, 2013).

It is therefore possible to argue that the difference between Acheulean populations in the south-west (with occasional examples of non-handaxe assemblages) and non-handaxe populations in central and eastern Europe can be substantiated. Non-handaxe assemblages within the south and west of Europe could be evidence of similar incursions by non-handaxe populations into the region. All of these sites share a common baseline technology and the only distinctive feature they share is the lack of handaxe manufacture.

The repetition of the pattern from MIS 11 and MIS 9 requires some level of explanation. Mithen’s (1994) work connecting the Clactonian to the effects of environmental change on group size and social learning would fit with a cyclical occurrence of non-handaxe assemblages. However, the model as it was originally constructed has been criticised due to the fact that the temperate environments argued to be the cause of Clactonian assemblages are noted as showing evidence for handaxe and non-handaxe sites (McNabb & Ashton, 1995; Wenban-Smith, 1998; White, 2000; Pettitt & White, 2012). This remains a fundamental problem, but the social role in technology and influence of group dynamics remains something to be explored further.

Wenban-Smith’s (2013) argument for an in situ development of the Acheulean within MIS 11 would require the same mechanism to have repeated in MIS 9. That would need some further explanation of the Acheulean being immanent within the Clactonian or the same drivers being present to account for the repeated re-invention of handaxes. Suggestions for this include changing raw materials or animal resources which encouraged behaviour to switch from an ad hoc expedient technology to one with more forward planning in the landscape (Wenban-Smith, 2013). It is not clear why this change in technology would be necessary, and how handaxes would be a functional improvement. Shipton (2020) has also argued that the handaxe was difficult to invent or emulate. It is later Middle Palaeolithic technology (most notably Levallois) which is often associated with higher levels of curation in the landscape (Geneste, 1985, 1989; Féblot-Augustins, 1999; White & Ashton, 2003; Scott, 2011; Scott et al., 2019). With the increasing evidence for the cultural importance of handaxes (White et al., 2018, 2019) and that non-handaxe assemblages may not represent cultures devoid of more complex behaviours (see Discussion), the argument for an in situ development of handaxes from the Clactonian seems rooted in simplistic evolutionary explanations. The flow of populations from source to sink regions in Europe (Dennell et al., 2011) seems more likely than the convergent evolution of the handaxe (White, 2023).

More plausibly, Ashton et al. (2016) suggested that across Europe a mosaic of cultural groupings existed, influenced by local circumstance with only smaller-scale drift during stable environments. The transitions from glacial to interglacial conditions during both MIS 12/11 and MIS 10/9 could therefore have caused the larger-scale movements of populations of hominins leaving refugia in the south of Europe, namely Iberia, Italy and the Balkans, after glacial periods, possibly even from outside Europe (Dennell et al., 2011). It is also possible that populations adapted to local circumstances, including a paucity of good-quality raw material, on the routes into northern Europe. One such route would be that used by groups coming from south-east Europe through central Europe in areas where evidence of handaxe manufacture is lacking. This reoccurrence in two successive cycles could be explained by more stable environments in south-western Europe, with central and eastern European populations being more affected by the amelioration of climate with major rivers (e.g. Danube, Rhine and Elba) creating corridors via which populations could have rapidly expanded into northern Europe. White and Schreve (2000) offered the possibility of physical or ecological barriers that may have affected hominin populations. The nature of Britain and its changing relationship to the continent, related to the rise and fall of sea level and its effect on British insularity, is one possible explanation for why these non-handaxe signatures are more visible and time constrained in comparison to the rest of western Europe where these subtle differences could be lost. The difficulty of entering and surviving in Britain possibly led to infrequent and short occupations which left distinctive cultural signals that have not been mixed (Ashton & Davis, 2021).

### The Significance of Non-handaxe Assemblages

The technological evidence from MIS 9 non-handaxe contexts fits with previous views that cores and flakes from non-handaxe assemblages form part of a baseline technology of knapping that is present in all hominin groups using hard-hammer technology (McNabb, 2007; Cole, 2011; Fluck, 2011). However, whilst this has been used to dismiss non-handaxe assemblages as lacking cultural significance, such arguments rather miss the point, especially given the increasing evidence for handaxe making being driven by cultural behaviours (Bridgland & White, 2014; Shipton, 2019a, b; White et al., 2019; Shipton & White, 2020; White, 2023; Dale et al., 2024). It can be argued that there are no distinctive forms of technology that unite non-handaxe sites in MIS 9, such as forms of flake tools or cores. However, unless reasons for the lack of handaxes at these sites can be found (raw material, functional), then it shows the occurrence of hominin groups that did not habitually make handaxes as part of their culture, and therefore differed from those groups for which handaxe manufacture was part of an inherited tradition.

Shipton (2010, 2019a, b) has argued that, whilst Oldowan technology is the result of emulation, the Acheulean resulted in clear over-imitation and a shared intentionality. The idea of a hominin lineage/species that lacked the capacity for social learning typically seen in Acheulean populations was posited by McNabb (2020). This seems untenable given evidence of other complex behaviour associated with non-handaxe assemblages (see in the following) and it could be argued that social norms differed and perhaps were not preserved in the archaeological record. For some, the Clactonian/non-handaxe assemblages are struggling to shake off the image of a ‘primitive’ form of technology linked with early hominin groups. Additionally, with the increasing variation detected in the hominin record of the Middle Pleistocene, traditional dichotomies between distinct species seem an unconvincing explanation for the absence/presence of handaxes (Dennell et al., 2011; Galway-Witham et al., 2019; Grün & Stringer, 2023).

The absence of handaxes in non-handaxe assemblages does not rule out other forms of behavioural complexity, which could have been culturally significant. There is evidence, as with handaxe sites, of use of less-durable materials, such bone tools (Julien et al., 2015; van Kolfschoten et al., 2015; Moigne et al., 2016; Zutovski & Barkai, 2016; Parfitt et al., 2022), wooden tools (Warren, 1911; Thieme, 1997; Schoch et al., 2015) and equally rare evidence for fire use (Mania, 1995; Gowlett et al., 2005; Preece et al., 2006; Roebroeks & Villa, 2011; de Lumley et al., 2015; Ravon et al., 2016a, b; Sanz et al., 2020). The best preserved evidence of Palaeolithic wooden technology in Britain comes from Clacton, which has previously led to speculation about the importance of wooden technology and other materials during the Clactonian (Warren, 1911).

More recently at Schöningen, evidence for wooden throwing spears has been found within a non-handaxe assemblage (Serangeli et al., 2018, 2023; Milks et al., 2023), along with bone tools, which are also found at Bilzingsleben, another German non-handaxe site (Mania & Mania, 2005). Whilst handaxes are absent from these contexts, there is evidence of hominins exploiting a wide range of material similar to these at handaxe sites. Evidence from Italy also demonstrates bone industries both with and without handaxes (Villa et al., 2016; Marinelli et al., 2024). Recently, Parfitt et al. (2022) have argued that use of bone soft hammers at Clacton shows a behaviourally more complex Clactonian than usually ascribed to non-handaxe assemblages. It should be noted that preservation is key in all these examples, due to specific taphonomic environments, and it should therefore not be argued that these materials were more important in non-handaxe producing groups than in those that produced handaxes. Rather, it demonstrates that arguing non-handaxe assemblages are a sign of hominin groups that lacked higher levels of social learning cannot be substantiated, and the significance lies in the fact that the handaxe-making tradition was simply not part of their repertoire.

These examples demonstrate the dangers of thinking of the relation between Clactonian and Acheulean in the evolutionary terms implied by the Mode system (Clark, 1969). Therefore, whilst handaxes have been linked to culture, normative behaviour and social cohesion (Shipton & White, 2020; Ashton & Davis, 2021; White, 2023), there is no reason to think these behaviours were not present in non-handaxe making groups. They may just not be visible due to preservation.

White (2023) stated that the Palaeolithic record of Britain shows long-term traditions. Non-handaxe sites from MIS 9 show one of these long-term traditions according to which handaxes are not made. This links with the idea of the Cultural Mosaic Model and the occurrence of localised traditions. There is therefore no need for a direct overarching link to the Clactonian. Although the possibility of a cultural relationship remains, the potential origin could derive from source populations in South-East Europe. This would fit in with regional and local differences that have begun to be identified in Europe (White et al., 2019; García-Medrano et al., 2023).

Intriguingly, Ashton and Davis’s (2021) ‘Assemblage Type 2’, which is defined by the presence of refined scrapers with invasive retouch on hard hammer flakes without handaxes during MIS 13 (typified at High Lodge), could be seen as another period of Lower Palaeolithic technology without handaxes. But in contrast with Clactonian and MIS 10/9 non-handaxe assemblages, it is not defined solely on the absence of handaxes but also by the presence of distinctive scrapers. Although evidence for this is lacking outside of the Brecklands, Stileman et al. (2024) suggested that these refined scrapers are a form of large cutting tool (LCT) and could show the use of mental templates (Hosfield, 2013). Experimental work by Stileman et al. (2024) points to soft hammer work being used outside of handaxe manufacture, as also shown by Parfitt et al. (2022) for Clacton, which again shows that rather than being a cruder technology, non-handaxe assemblages could be alternative expressions of Lower Palaeolithic culture. This is evidence that non-handaxe sites are not part of one overarching Clactonian culture but are still vital to understanding more nuanced patterns in the Lower Palaeolithic.

## Conclusion

The evidence for a non-handaxe signature in MIS 10/9 can be substantiated at three non-handaxe sites in the Thames and its tributaries and at the site of Redhill in East Anglia. However, further sites, previously claimed to contain ‘Clactonian elements’, must be rejected on the current evidence. Whilst there are reservations about the non-handaxe assemblages (McNabb, 2020), our current interpretation of their dating and technology is that, similar to MIS 11, there is a non-handaxe signature during MIS 10/9 that is evidence of the presence of hominins who did not habitually make handaxes. Despite this, there is no evidence that the technology is distinct from wider Lower Palaeolithic core-and-flake working, and therefore it cannot be linked to an overarching Clactonian.

When contextualised within the European evidence, it is clear that both the Clactonian and the MIS 9 non-handaxe signature relate to a wider trend across the Middle Pleistocene of Europe. The significance of non-handaxe assemblages is still debated but the chronological patterning in the British Palaeolithic seems to dismiss raw material or functional explanations, and a cultural reason seems the most apt. The social role handaxes played in the lives of hominins suggests that their absence must have had a distinct cultural reason. The cultural mosaics model (Ashton & Davis, 2021) and Dennell et al.’s (2011) work provide clear mechanisms for behavioural variability within the Lower Palaeolithic, and work in Britain is beginning to reveal these distinct assemblage types.

This should be regarded as a working hypothesis. Future work on MIS 9 sites is important either to strengthen or disprove this position. The recent work in the Brecklands (Davis et al., 2024) demonstrates how new work can help towards answering longstanding questions, such as evidence that this non-handaxe signature extends beyond the Thames and its tributaries, as the Clactonian does at Barnham (Ashton et al., 2016). Future work at the site of Biddenham could potentially be insightful, but caution is needed when using old collections without clear provenance unless archival work or new fieldwork can add further context. Obviously, new work on these sites uncovering evidence of handaxe manufacture or changing our current understanding of other aspects could falsify this working hypothesis. Furthermore, the discovery of non-handaxe sites outside of the late MIS 10/early MIS 9 (White & Schreve, 2000) would also re-open questions surrounding other explanations for non-handaxe sites, as suggested for a number of European sites.

It is notable that three of the five ‘flagship’ sites discussed by White and Bridgland (2018) contain evidence for a non-handaxe signature at the beginning of the interglacial. Whilst these lack the level of evidence akin to that from MIS 11, it is imperative that the MIS 9 non-handaxe signature is continued to be treated seriously. As previously stated by White (2000), given the need for large primary-context assemblages, it is not surprising that there is only a small number of accepted non-handaxe sites in Britain.

## Supplementary Information

Below is the link to the electronic supplementary material.

Supplementary file1 (DOCX 31 KB)

Supplementary file2 (DOCX 21 KB)

Supplementary file3 (DOCX 118 KB)

## Data Availability

The materials can be accessed at the following institutions (all in the UK): The British Museum, The Higgins Bedford, The Pitt Rivers Museum, Oxford Natural History Museum, Cambridge Archaeology and Anthropology Museum, Reading Museum, Hampshire Cultural Trust, Sedgewick Museum, Portsmouth Museum, and Royal Holloway, University of London.
