# Supplementary material for: The Occurrence of Non-handaxe Assemblages Early in the Purfleet Interglacial (MIS 9) in Britain
Source: J Paleolit Archaeol. 2025 May 17;8(1):18. doi: 10.1007/s41982-025-00217-2 (PMC12085398; doi:10.1007/s41982-025-00217-2)
Supplement: Supplementary file 1 — Supplementary file1 (DOCX 31 KB) [file 41982_2025_217_MOESM1_ESM.docx]

**SOM 1 MIS 9 site details**

SOM1 Table 1 Summary of Purfleet sequence (Schreve et al., 2002; Bridgland et al., 2013 ; White et al., 2024).

| Bed #/name | Thickness | Archaeology | Pit | Environment |
| --- | --- | --- | --- | --- |
| 8. Botany Gravel | 2m | Acheulean. Previously argued to contain distinct Proto-Levallois/Levallois (cf.White et al., 2024) | Greenlands, Bluelands, Botany | Cold? |
| 7. Grey-brown silty clay, weathered | <0.75m |  | North side of Greenland quarry and in the Esso Sport Field boreholes | Temperate? |
| 6. Bluelands Gravel | Up to 6m | Acheulean | Bluelands, Greenlands, Esso, Botany | Cold? |
| 5. Greenlands Shell Bed | Up to 2m | ? a few flakes | Greenlands, Bluelands | Temperate. Contains an abundance of temperate shells, most articulated in life-position, as well as thermophilous fish and an interglacial mammalian fauna. A range of local marshland, woodland and grassland, and slow to fast moving aquatic habitats are indicated, including a weak brackish influence |
| 4. Laminated Silty Clay | <0.25m |  | Greenlands,Bluelands | Temperate. Possibly tidal sedimentation during high sea levels. Temperate ostracods , some with brackish tolerances. Mosaic open woodland with 70% temperate arboreal pollen including oak, ash, lime, elm and spruce. Remains of green frog suggest mean July temperatures ~15-17˚C |
| 3. Shelly Gravel | <0.75m | Non-handaxe (cf. Clactonian) | Greenlands,Bluelands | Temperate |
| 2. Little Thurrock Gravel | <0.4m | Non-handaxe (cf. Clactonian) | Greenlands,Bluelands | Cold |
| 1. Angular chalk rubble (Coombe Rock) lying on Chalk | 1m | Non-handaxe (cf. Clactonian) | Greenlands,Bluelands | Cold |

SOM1 Table 2 Summary of key MIS 9 sites.

| Site | Dating | Archaeology | Context | Key references |
| --- | --- | --- | --- | --- |
| Purfleet | MIS 10–9–8 | 2 main assemblages:  1) Non-handaxe from Little Thurrock Gravels. 2) Acheulean assemblage from Bluelands Gravels and Botany Gravels.  Previously argued distinct proto-Levallois/Levallois from Bluelands/ Botany Gravels (cf White et al., 2024) | Long sequence through the Lynch Hill/Corbets Tey Formation of the Thames (SOM 1). | Schreve et al., 2002; Bridgland et al., 2013 ; White et al., 2024 |
| Globe Pit, Little Thurrock | Late MIS 10  /Early MIS 9 | Non-handaxe assemblage containing numerous flakes and cores. | Basal part of Lynch Hill/Corbets Tey Formation of the Thames.  Lateral equivalent of overlying brickearth (Grays Brickearth) contains MIS 9 fauna. | Bridgland and Harding, 1993; Bridgland, 1994 |
| Stoke Newington | MIS 9 | In situ ‘floor’ containing handaxes, débitage, scrapers and cores. Conjoins present. Rolled artefacts occurred lower down the sequence.  Roe’s Group I. | 2–3m of fine sand at the confluence of the Lea and Thames.  Represents a part of MIS 9 that predates the organic deposits at the Nightingale Estate, Hackney, although the age difference is probably minor. | Green et al., 2004; 2006 |
| Wolvercote Channel | MIS 9 | Acheulean assemblage with subgroup of plano-convex ‘slipper-shaped’ handaxes.  Roe’s Group III. | 4.5m deep sediment-filled channel cut into Wolvercote Terrace Gravel on the west bank of the Upper Thames, south of its confluence with the River Cherwell. | Tyldesley, 1986; Bridgland, 1994 |
| Cuxton | MIS 9–8 | 2 main assemblages:  1)Non-handaxe assemblage from lower gravel (layers 1-6)  2)Acheulean assemblage from upper gravel, includes ficrons, cleavers  Roe’s Group I (layers 7+). | Remnant of Medway terrace gravel, situated on a Chalk spur between the Medway and a tributary valley.  Bridgland (2003) proposed an MIS 10/9/8 date for the Cuxton sequence, a suggestion supported by recent OSL dates that gave an absolute age compatible with an MIS 8 age for the Acheulean material; OSL determinations which indicate an MIS 7 age have been considered too. | Tester, 1965; Cruse, 1987; Bridgland, 2003; Wenban-Smith, 2004 |

SOM1 Table 3 Summary of secondary context sites correlated to MIS 9.

| **System** | **Area** | **Sites** | **MIS** | **Context** | **Main collectors** | **References** |
| --- | --- | --- | --- | --- | --- | --- |
| Thames | Maidenhead | Pits in Furze Platt area including Cannoncourt Farm | 10–9–8 | Lynch Hill Terrace.  4m of bedded gravel, overlain by a pebbly, silty clay. | Treacher, Lacaille | Wymer, 1968; Bridgland, 1994 |
|  | Farnham Royal | Baker’s Farm | 10–9–8 | Lynch Hill Terrace.  Similar to Furze Platt. Ill-sorted fluvial gravels overlying Reading beds.  Artefacts associated with lowest part of stratified gravels. | Treacher, Lacaille | Wymer, 1968 |
|  | Burnham | Lent Rise | 10–9–8 | Lynch Hill Terrace.  Similar to Furze Platt. Ill-sorted but stratified gravel overlain by brickearth. | Lacaille | Wymer, 1968 |
|  | Reading | Grovelands Pit | 10–9–8 | Lynch Hill Terrace. Bluff gravel between the Lynch Hill and Taplow terraces.  4 meters of gravel underlain by sand and clay. | Treacher | Wymer, 1968 |
| Solent | Dunbridge | Several pits covering two terraces | 10-9-8 | Terrace 2/3 of the Test.  Two gravel terraces: Upper Belbin Formation and a Lower Mottisont Formation. | Local collectors; Dale; Harding and Bridgland | Harding et al., 2012 ; Davis et al., 2021 |
|  | Warsash | Several pits | 10–9–8 | Top of Terrace 3 of Test.  Varying thickness of gravel across four pits (New, Park, Dykes, and Newbury). | Codrington; Draper; Mogridge | Westaway et al., 2006; Davis et al., 2016; Hatch et al., 2017 |
| Great Ouse | Biddenham/Kempston | Several pits | 10–9–8 | Terrace 3 gravels, sands and silts  ‘Biddenham member’, at 14.5-18m OD.  Archaeology associated with organic beds with rich temperate signatures. | WG Smith; Wyatt | Harding et al., 1991 Boreham et al., 2010 |
| Little Ouse | Barnham Heath | Newport’s Pit | 11–9 | 6-8m above the flood plain.  5.8m of sandy gravels resting on disturbed chalk with archaeology coming from the base. | Brown | Wymer, 1985 |
|  | Redhill (basal gravel) | Several pits (Breckland Palaeolithic Project) | 10-9-8 (10-9) | Redhill terrace Basal gravel- 1.6 m thick coarse chalk-rich flint gravel, with a grey chalky sand matrix, fine to very coarse sub-rounded to angular flint clasts. | Breckland Palaeolithic Project | Davis et al., 2024 |
| Kennett | Kennett/Kentford | Station Pit | 10–9–8 | Terrace 3.  4-5m of gravel well bedded gravel. | Wright and Whitaker | Boreham et al., 2010 |

Boreham, S., White, T.S., Bridgland, D.R., Howard, A.J., White, M.J., 2010. The Quaternary history of the Wash fluvial network, UK. Proc. Geol. Assoc. 121, 393-409.

Bridgland, D. R. and Harding, P. 1993. ‘Middle Pleistocene Thames terrace deposits at Globe Pit, Little Thurrock, and their contained Clactonian industry’, Proceedings of the Geologists’ Association, 104. 263–283.

Bridgland, D.R., Harding, P., Allen, P., Candy, I., Cherry, C., George, W., Horne, D.J., Keen, D.H., Penkman, K.E.H., Preece, R.C., Rhodes, E.J., Scaife, R., Schreve, D.C., Schwenninger, J., Slipper, I., Ward, G.R., White, M.J., White, T.S., Whittaker, J.E., 2013. An enhanced record of MIS 9 environments, geochronology and geoarchaeology: data from construction of the High Speed 1 (London–Channel Tunnel) rail-link and other recent investigations at Purfleet, Essex, UK. Proc. Geol. Assoc. 124, 417-476.

Bridgland, D.R., 1994. Quaternary of the Thames. Chapman and Hall, London.

Bridgland, D.R., 2003. The evolution of the River Medway, SE England, in the context of Quaternary palaeoclimate and the Palaeolithic occupation of NW Europe. Proc. Geol. Assoc. 114, 23-48.

Bridgland, D.R., 2006. The Middle and Upper Pleistocene sequence in the Lower Thames: A record of Milankovitch climatic fluctuation and early human occupation of southern Britain: Henry Stopes Memorial Lecture. Proc. Geol. Assoc. 117, 281–305.

Cruse, R., Bridgland, D., Callow, P., Currant, A., Hubbard, R., Debenham,N., Bowman, S., 1987. Further Investigation of the Acheulian Site at Cuxton. Archaeol. Cantiana 104, 39-81.

Davis, R., Ashton, N., Hatch, M., Hosfield, R. and Lewis, S.G., 2021. Lower and early middle Palaeolithic of southern Britain: The evidence from the River Test. Journal of Paleolithic Archaeology, 4, pp.1-50.

Davis, R, J., Hatch, M., Ashton, N., Hosfield, R., Lewis, S., 2016. The Palaeolithic record of Warsash, Hampshire, UK: Implications for late Lower and early Middle Palaeolithic occupation history of Southern Britain. Proc. Geol. Assoc. 127, 558–574.

Davis, R., Lewis, S., Hatch, M., Ashton, N., Voinchet, P., Bahain, J.J., Dale, L., Foulds, F., Rawlinson, A. and White, M., 2024. A revised terrace stratigraphy and chronology for the Little Ouse River as a framework for interpreting the late Lower and early Middle Palaeolithic of central East Anglia, UK. *Quaternary Environments and Humans*, p.100045.

Green, C., Branch, N., Coope, G., Field, M., Keen, D., Wells, J., Schwenninger, J Preece, R., Schreve, D., Canti, M. and Gleed-Owen, C. 2006. ‘Marine Isotope Stage 9 environments of fluvial deposits at Hackney, north London, UK’, Quaternary Science Reviews, 25. 89–113.

Green, C., Gibbard, P., Bishop, C., 2004. Stoke Newington: geoarchaeology of the Palaeolithic ‘floor’. Proc. Geol. Assoc. 115, 193-207.

Harding, P., Bridgland, D., Allen, P., Bradley, P., Grant, M., Peat, D., Schwenninger, J., Scott, R., Westaway, R., White, T., 2012. Chronology of the Lower and Middle Palaeolithic in NW Europe: Developer-funded investigations at Dunbridge, Hampshire, southern England. Proc. Geol. Assoc. 123, 584-607.

Harding, P., Bridgland, D., Keen, D., Rogerson. R., 1991. A Palaeolithic site rediscovered at Biddenham, Bedfordshire. Bedfordshire Archaeology 19, 7-90.

Hatch, M., Davis, R., Lewis, S., Ashton, N., Briant, R., Lukas, S., 2017. The stratigraphy and chronology of the fluvial sediments at Warsash, UK: Implications for the Palaeolithic archaeology of the River Test Proc. Geol. Assoc. 128, 198-221.

Roe, D., 1968b. British Lower and Middle Palaeolithic handaxe groups. Proc. Prehist. Soc. 34, 1–82.

Schreve, D.C., Bridgland, D.R., Allen, P., Blackford, J.J., Gleed-Owen, C.P., Griffiths, H.I., Keen, D.H., White, M.J., 2002. Sedimentology, palaeontology and archaeology of late Middle Pleistocene River Thames terrace deposits at Purfleet, Essex, UK. Quat. Sci. Rev. 21, 1423-1464.

Tester, P. J., 1965. An Acheulian site at Cuxton. Archaeologia Cantiana 80, 20-60.

Tyldesley, J.A. 1986. The Wolvercote Channel Handaxe Assemblage: a comparative study. Oxford, British Archaeological Report, 153.

Wenban-Smith, F., 2004. Handaxe typology and Lower Palaeolithic cultural development: Ficrons, cleavers and two giant handaxes from Cuxton. Lithics 25, 11-21.

Westaway, R.C., Bridgland, D.R., White, M.J., 2006 The Quaternary uplift history of central southern England: evidence from the terraces of the Solent River system and nearby raised beaches. Quat. Sci. Rev. 25, 2212-2250.

White, M. J. 2000. ‘The Clactonian Question: on the interpretation of core and flake assemblages in the British Isles’, *Journal of World Prehistory, 14*. 1-63.

White, M., Rawlinson, A., Foulds, F., Dale, L., Davis, R., Bridgland, D., Shipton, C. and Ashton, N., 2024. Making a U-turn on the Purfleet Interchange: Stone Tool Technology in Marine Isotope Stage 9 Britain and the Emergence of the Middle Palaeolithic in Europe. Journal of Paleolithic Archaeology, 7(1), pp.1-23.

White, M.J., Ashton, N., 2003. Lower Palaeolithic core technology and the origins of the Levallois method in north-western Europe. Curr. Anthropol. 44, 598-609.

White, M.J., Bridgland, D.R., 2018. Thresholds in lithic technology and human behaviour in MIS 9 Britain. In: Pope, M., McNabb, J., Gamble, C. (Eds.), Crossing the Human Threshold: Dynamic Transformation and Persistent Places during the Middle Pleistocene. Routledge, London, pp.165-192.

Wymer, J., 1968. Lower Palaeolithic of Britain, as Represented by the Thames Valley. John Baker, London.

Wymer, J., 1985. Palaeolithic Sites of East Anglia. Geobooks, Norwich.
