# Supplementary material for: The Occurrence of Non-handaxe Assemblages Early in the Purfleet Interglacial (MIS 9) in Britain
Source: J Paleolit Archaeol. 2025 May 17;8(1):18. doi: 10.1007/s41982-025-00217-2 (PMC12085398; doi:10.1007/s41982-025-00217-2)
Supplement: Supplementary file 2 — Supplementary file2 (DOCX 21 KB) [file 41982_2025_217_MOESM2_ESM.docx]

SOM 2 Artefact analysis methodology

Condition

Recorded on a three point scale:

1) Fresh

2) Slightly Rolled

3) Rolled

Flakes

The following attributes were recorded:

**Length, width and thickness of flakes**- Recorded in mm with length being measured parallel to the axis of percussion and width perpendicular to the axis of percussion.

**Broken/type of break**- Recorded to indicate either knapping or post-depositional breaks.

**Cortex**- The percentage of cortex left on the dorsal surface was recorded to relate the flake to its stage in the knapping sequence.

**Flake type**- Recorded as a judgment of the stage of knapping the flake belonged to (after Ashton, 1998):

1. Cortical surface and butt.
2. Either >50% cortical dorsal surface and cortical butt or cortical surface and non-cortical butt.
3. Either <50% cortical dorsal surface and cortical butt or >50% cortical dorsal surface and non-cortical butt.
4. Either non-cortical dorsal surface and cortical butt, or <50% cortical surface and non-cortical butt.
5. Non-cortical dorsal surface and non-cortical butt.

**Dorsal scar count**- Previous removals identified from the dorsal side of the flake.

**Dorsal scar pattern**- Simplified from the methodologies of Ashton (1998) and Ashton and McNabb (1996). These categories relate to how the core that produced the flake was worked, focussing on the complexity of the patterns:

- Unidirectional
- Bidirectional
- Multidirectional
- Natural or cortical

**Butt type**- The nature of the butt was recorded to assess the type of percussion, preparation and technology:

1. Plain- formed from part of a single flake scar.
2. Dihedral- formed at the intersection of two or more flake scars.
3. Cortical- covered in cortex.
4. Natural- natural surface without cortex.
5. Marginal- formed at the edge of a core forming a narrow, indeterminate butt.
6. Mixed- formed from a combination of cortical/natural and flake scars.
7. Faceted- Shows evidence of preparation.
8. Missing- butt not present due to broken flake.

Cores

**Length, width and thickness-** Recorded in (mm).

**Typology**- Cores were placed in one of the following broad categories (Scott, 2011):

- Migrating platform cores (MPC)- generic term for undiagnostic cores made up of core episodes, with minimal organisation.
- Discoidal- Core divided by plane of intersection but not hierarchically.
- Chopper cores- evidence of modification to one edge opposite a cortical ‘grip’.
- Proto-Levallois
- Levallois

**Core episodes**- The sequences of working were interpreted, and the cores were divided into reduction episodes (Ashton and McNabb, 1996). This process interrogates the history of the core rather than focusing on a static end shape (Ashton, 1998).

 Reduction episodes were characterised as follows:

- Single removal, Type A- Single removal from the surface of the core, linked to other core episodes.
- Parallel flaking, Type B- Two or more removals in a parallel direction from the same or adjacent platform.
- Alternative flaking, Type C – One or more removals form the platform or platforms of the next set of removals. The core is turned at least once but could be turned multiple times.
- Unrelated single removal, Type D- Single removal that cannot be associated with any other removal.

Flake tools

The following attributes were recorded from all retouched flakes in line with the methodologies of Inizan et al*.* (1999) and Scott (2011).

**Quantitative measurements**

Length, width and thickness of flake tools- Recorded in mm.

Elongation was calculated from these measurements by dividing width by length.

Length of retouch- Recorded in mm.

Length of retouch was also calculated as a proportion of artefact length.

**Qualitative observations**

Typology: Flake tools were placed into categories based on their characteristics.

Position of retouch was recorded as an assessment of whether retouch was confined to one side of the artefact. Where only one side was retouched the relation to the dorsal surface was recorded:

- Direct- Retouch on the dorsal surface.
- Inverse- Retouch on the ventral surface.
- Alternate- Retouch on opposite edges on both faces.
- Bifacial- Retouch of both faces on the same edge.

Distribution of retouch was recorded as an assessment of whether the retouch was one sequence or unrelated ad hoc removals:

- Continuous
- Discontinuous

Regularity of retouch was recorded. While similar to distribution, this was an assessment of how uniform retouch was:

- Regular
- Irregular

Form of the retouched edge was recorded as an assessment of the shape the edge of the tool had been retouched into:

- Rectilinear
- Convex
- Concave
- Notch
- Denticulate

Invasiveness of retouch was recorded as an assessment of the extent the retouch on the surface of the flake tool:

- Minimally invasive
- Semi-invasive
- Invasive

Location of retouch was recorded as an assessment of where the flakes had been retouched:

- Distal
- Left
- Right
- Multiple
- Proximal

In order to confidently identify Levallois flakes, the criteria used by Scott (2011) were followed:

- Hard hammer percussion.
- Large number of dorsal scars, possibly in a complex pattern.
- Removed from the surface rather than the volume of the core, making the flake relatively flat.
- Signs of distal and lateral convexities being controlled.
- May retain evidence of faceting or other methods of platform preparation.
- May retain evidence of deliberate convexity accentuation, including small peripheral flake scars.

Ashton, N. 1998. ‘Flint analysis methodology’, In (eds.) N. Ashton S. Lewis S. Parfitt Excavations at the Lower Palaeolithic site at East Farm Barnham, Suffolk, 1989-94. London, British Museum Press. 288-292.

Ashton, N. and McNabb, J. 1996. ‘Methodology of flint analysis’, In (eds.) B. Conway J. McNabb and N. Ashton Excavations at Barnfield Pit, Swanscombe, 1968-72. Occasional Paper No.94. London, British Museum Press. 241-247.

Inizan, M., Reduron-Ballinger, M., Roche, M. and Tixier, J. 1999. Technology and terminology of knapped stone. Préhistoire de la pierre taillée 5, Éditions du Crep, Nanterre, France. Nanterre, CREP.

Scott, B. 2011. Becoming Neanderthals: The Earlier British Middle Palaeolithic. Oxford, Oxbow Books.
