# Supplementary material for: The Occurrence of Non-handaxe Assemblages Early in the Purfleet Interglacial (MIS 9) in Britain
Source: J Paleolit Archaeol. 2025 May 17;8(1):18. doi: 10.1007/s41982-025-00217-2 (PMC12085398; doi:10.1007/s41982-025-00217-2)

SOM 3: Statistical tests of metrics from MIS 9 flakes

An ANOVA test indicated that there was a statistically significant difference between flake length, width, thickness and elongation across the sites within the study.


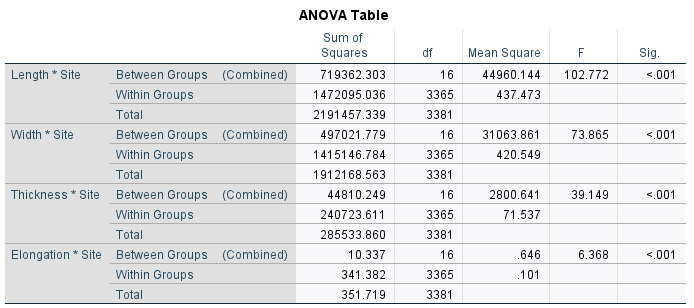


A TukeyHSD post-hoc analysis was then used to determine pairwise differences in flake length across the different sites. Sites were often significantly different, with exceptions in bold. The analysis supports the idea that the metrics reflect the method of collection with excavated or carefully collected handaxe assemblages such as Cuxton and Stoke Newington being closer to the excavated non-handaxe assemblages. The Redhill assemblage shows similarities to a broad range of sites. This is discussed in the main text as being due to the presence of more intensive working and low representation of earlier stages of working similar to the collected samples.

| **Multiple Comparisons** | | | | | | |
| --- | --- | --- | --- | --- | --- | --- |
| Dependent Variable: Length | | | | | | |
| Tukey HSD | | | | | | |
| (I) Site | (J) Site | Mean Difference (I-J) | Std. Error | Sig. | 95% Confidence Interval | |
|  |  |  |  |  | Lower Bound | Upper Bound |
| Baker's Farm | Barnham Heath | -18.5686^*^ | 1.92936 | <.001 | -25.2464 | -11.8909 |
|  | **Biddenham** | **7.3776^*^** | **1.72912** | **.002** | **1.3929** | **13.3623** |
|  | Cuxton 1-6 | 20.5679^*^ | 2.50369 | <.001 | 11.9023 | 29.2334 |
|  | Cuxton 7+ | 30.4209^*^ | 2.32320 | <.001 | 22.3800 | 38.4617 |
|  | Cuxton Tester | 21.4922^*^ | 1.79023 | <.001 | 15.2960 | 27.6884 |
|  | **Dunbridge** | **.4924** | **2.54746** | **1.000** | **-8.3247** | **9.3094** |
|  | Furze Platt | 8.4776^*^ | 1.89890 | .001 | 1.9053 | 15.0499 |
|  | Globe Pit | 33.8993^*^ | 1.69319 | <.001 | 28.0390 | 39.7596 |
|  | **Groveland's Pit** | **-9.8041^*^** | **2.51216** | **.011** | **-18.4989** | **-1.1092** |
|  | **Kempston** | **1.6380** | **2.44060** | **1.000** | **-6.8092** | **10.0852** |
|  | **Kentford** | **-3.1022** | **2.28474** | **.996** | **-11.0100** | **4.8055** |
|  | **Lent Rise** | **6.0107** | **2.55666** | **.616** | **-2.8382** | **14.8596** |
|  | Purfleet | 20.2053^*^ | 3.45284 | <.001 | 8.2546 | 32.1560 |
|  | **Redhill (B. Gravel)** | **10.1469** | **3.52073** | **.244** | **-2.0388** | **22.3325** |
|  | Stoke Newington | 15.1734^*^ | 1.73047 | <.001 | 9.1841 | 21.1628 |
|  | **Warsash** | **1.6312** | **2.83822** | **1.000** | **-8.1922** | **11.4546** |
| Barnham Heath | Baker's Farm | 18.5686^*^ | 1.92936 | <.001 | 11.8909 | 25.2464 |
|  | Biddenham | 25.9462^*^ | 1.65929 | <.001 | 20.2032 | 31.6892 |
|  | Cuxton 1-6 | 39.1365^*^ | 2.45599 | <.001 | 30.6360 | 47.6369 |
|  | Cuxton 7+ | 48.9895^*^ | 2.27171 | <.001 | 41.1268 | 56.8521 |
|  | Cuxton Tester | 40.0609^*^ | 1.72288 | <.001 | 34.0978 | 46.0240 |
|  | Dunbridge | 19.0610^*^ | 2.50059 | <.001 | 10.4062 | 27.7158 |
|  | Furze Platt | 27.0462^*^ | 1.83554 | <.001 | 20.6932 | 33.3992 |
|  | Globe Pit | 52.4679^*^ | 1.62182 | <.001 | 46.8546 | 58.0812 |
|  | **Groveland's Pit** | **8.7646^*^** | **2.46462** | **.037** | **.2342** | **17.2949** |
|  | Kempston | 20.2066^*^ | 2.39164 | <.001 | 11.9289 | 28.4844 |
|  | Kentford | 15.4664^*^ | 2.23237 | <.001 | 7.7399 | 23.1929 |
|  | Lent Rise | 24.5793^*^ | 2.50996 | <.001 | 15.8920 | 33.2666 |
|  | Purfleet | 38.7739^*^ | 3.41840 | <.001 | 26.9424 | 50.6054 |
|  | Redhill (B. Gravel) | 28.7155^*^ | 3.48697 | <.001 | 16.6467 | 40.7843 |
|  | Stoke Newington | 33.7420^*^ | 1.66070 | <.001 | 27.9941 | 39.4899 |
|  | Warsash | 20.1998^*^ | 2.79623 | <.001 | 10.5217 | 29.8779 |
| Biddenham | **Baker's Farm** | **-7.3776^*^** | **1.72912** | **.002** | **-13.3623** | **-1.3929** |
|  | Barnham Heath | -25.9462^*^ | 1.65929 | <.001 | -31.6892 | -20.2032 |
|  | Cuxton 1-6 | 13.1903^*^ | 2.30202 | <.001 | 5.2227 | 21.1578 |
|  | Cuxton 7+ | 23.0432^*^ | 2.10430 | <.001 | 15.7600 | 30.3265 |
|  | Cuxton Tester | 14.1146^*^ | 1.49524 | <.001 | 8.9394 | 19.2898 |
|  | **Dunbridge** | **-6.8852** | **2.34954** | **.218** | **-15.0173** | **1.2468** |
|  | **Furze Platt** | **1.0999** | **1.62377** | **1.000** | **-4.5201** | **6.7200** |
|  | Globe Pit | 26.5217^*^ | 1.37757 | <.001 | 21.7538 | 31.2896 |
|  | Groveland's Pit | -17.1817^*^ | 2.31122 | <.001 | -25.1811 | -9.1823 |
|  | **Kempston** | **-5.7396** | **2.23324** | **.449** | **-13.4691** | **1.9899** |
|  | Kentford | -10.4798^*^ | 2.06176 | <.001 | -17.6158 | -3.3439 |
|  | **Lent Rise** | **-1.3669** | **2.35952** | **1.000** | **-9.5335** | **6.7997** |
|  | **Purfleet** | **12.8277^*^** | **3.30951** | **.012** | **1.3731** | **24.2823** |
|  | **Redhill (B. Gravel)** | **2.7693** | **3.38029** | **1.000** | **-8.9303** | **14.4688** |
|  | Stoke Newington | 7.7958^*^ | 1.42315 | <.001 | 2.8701 | 12.7215 |
|  | **Warsash** | **-5.7464** | **2.66202** | **.755** | **-14.9600** | **3.4671** |
| Cuxton 1-6 | Baker's Farm | -20.5679^*^ | 2.50369 | <.001 | -29.2334 | -11.9023 |
|  | Barnham Heath | -39.1365^*^ | 2.45599 | <.001 | -47.6369 | -30.6360 |
|  | Biddenham | -13.1903^*^ | 2.30202 | <.001 | -21.1578 | -5.2227 |
|  | **Cuxton 7+** | **9.8530^*^** | **2.77609** | **.038** | **.2446** | **19.4614** |
|  | **Cuxton Tester** | **.9244** | **2.34827** | **1.000** | **-7.2033** | **9.0520** |
|  | Dunbridge | -20.0755^*^ | 2.96631 | <.001 | -30.3422 | -9.8088 |
|  | Furze Platt | -12.0903^*^ | 2.43213 | <.001 | -20.5082 | -3.6724 |
|  | Globe Pit | 13.3314^*^ | 2.27515 | <.001 | 5.4569 | 21.2060 |
|  | Groveland's Pit | -30.3719^*^ | 2.93604 | <.001 | -40.5339 | -20.2099 |
|  | Kempston | -18.9298^*^ | 2.87506 | <.001 | -28.8808 | -8.9789 |
|  | Kentford | -23.6701^*^ | 2.74399 | <.001 | -33.1674 | -14.1728 |
|  | Lent Rise | -14.5572^*^ | 2.97421 | <.001 | -24.8513 | -4.2631 |
|  | **Purfleet** | **-.3626** | **3.77247** | **1.000** | **-13.4195** | **12.6944** |
|  | **Redhill (B. Gravel)** | **-10.4210** | **3.83470** | **.344** | **-23.6934** | **2.8513** |
|  | **Stoke Newington** | **-5.3945** | **2.30303** | **.623** | **-13.3655** | **2.5766** |
|  | Warsash | -18.9367^*^ | 3.21946 | <.001 | -30.0796 | -7.7937 |
| Cuxton 7+ | Baker's Farm | -30.4209^*^ | 2.32320 | <.001 | -38.4617 | -22.3800 |
|  | Barnham Heath | -48.9895^*^ | 2.27171 | <.001 | -56.8521 | -41.1268 |
|  | Biddenham | -23.0432^*^ | 2.10430 | <.001 | -30.3265 | -15.7600 |
|  | **Cuxton 1-6** | **-9.8530^*^** | **2.77609** | **.038** | **-19.4614** | **-.2446** |
|  | **Cuxton Tester** | **-8.9286^*^** | **2.15480** | **.004** | **-16.3866** | **-1.4706** |
|  | Dunbridge | -29.9285^*^ | 2.81563 | <.001 | -39.6737 | -20.1833 |
|  | Furze Platt | -21.9433^*^ | 2.24590 | <.001 | -29.7166 | -14.1700 |
|  | **Globe Pit** | **3.4785** | **2.07488** | **.963** | **-3.7029** | **10.6598** |
|  | Groveland's Pit | -40.2249^*^ | 2.78373 | <.001 | -49.8597 | -30.5901 |
|  | Kempston | -28.7828^*^ | 2.71933 | <.001 | -38.1948 | -19.3709 |
|  | Kentford | -33.5231^*^ | 2.58037 | <.001 | -42.4540 | -24.5921 |
|  | Lent Rise | -24.4102^*^ | 2.82396 | <.001 | -34.1842 | -14.6361 |
|  | **Purfleet** | **-10.2156** | **3.65518** | **.295** | **-22.8665** | **2.4354** |
|  | Redhill (B. Gravel) | -20.2740^*^ | 3.71938 | <.001 | -33.1472 | -7.4008 |
|  | Stoke Newington | -15.2474^*^ | 2.10541 | <.001 | -22.5345 | -7.9604 |
|  | Warsash | -28.7897^*^ | 3.08119 | <.001 | -39.4540 | -18.1253 |
| Cuxton Tester | Baker's Farm | -21.4922^*^ | 1.79023 | <.001 | -27.6884 | -15.2960 |
|  | Barnham Heath | -40.0609^*^ | 1.72288 | <.001 | -46.0240 | -34.0978 |
|  | Biddenham | -14.1146^*^ | 1.49524 | <.001 | -19.2898 | -8.9394 |
|  | **Cuxton 1-6** | **-.9244** | **2.34827** | **1.000** | **-9.0520** | **7.2033** |
|  | **Cuxton 7+** | **8.9286^*^** | **2.15480** | **.004** | **1.4706** | **16.3866** |
|  | Dunbridge | -20.9999^*^ | 2.39488 | <.001 | -29.2888 | -12.7109 |
|  | Furze Platt | -13.0147^*^ | 1.68870 | <.001 | -18.8595 | -7.1699 |
|  | Globe Pit | 12.4071^*^ | 1.45354 | <.001 | 7.3762 | 17.4379 |
|  | Groveland's Pit | -31.2963^*^ | 2.35729 | <.001 | -39.4552 | -23.1374 |
|  | Kempston | -19.8542^*^ | 2.28088 | <.001 | -27.7486 | -11.9598 |
|  | Kentford | -24.5945^*^ | 2.11328 | <.001 | -31.9088 | -17.2802 |
|  | Lent Rise | -15.4815^*^ | 2.40467 | <.001 | -23.8044 | -7.1587 |
|  | **Purfleet** | **-1.2869** | **3.34185** | **1.000** | **-12.8535** | **10.2796** |
|  | **Redhill (B. Gravel)** | **-11.3454** | **3.41195** | **.076** | **-23.1545** | **.4638** |
|  | **Stoke Newington** | **-6.3188^*^** | **1.49681** | **.003** | **-11.4994** | **-1.1382** |
|  | Warsash | -19.8611^*^ | 2.70211 | <.001 | -29.2134 | -10.5087 |
| Dunbridge | **Baker's Farm** | **-.4924** | **2.54746** | **1.000** | **-9.3094** | **8.3247** |
|  | Barnham Heath | -19.0610^*^ | 2.50059 | <.001 | -27.7158 | -10.4062 |
|  | **Biddenham** | **6.8852** | **2.34954** | **.218** | **-1.2468** | **15.0173** |
|  | Cuxton 1-6 | 20.0755^*^ | 2.96631 | <.001 | 9.8088 | 30.3422 |
|  | Cuxton 7+ | 29.9285^*^ | 2.81563 | <.001 | 20.1833 | 39.6737 |
|  | Cuxton Tester | 20.9999^*^ | 2.39488 | <.001 | 12.7109 | 29.2888 |
|  | **Furze Platt** | **7.9852** | **2.47716** | **.102** | **-.5885** | **16.5589** |
|  | Globe Pit | 33.4069^*^ | 2.32323 | <.001 | 25.3660 | 41.4479 |
|  | **Groveland's Pit** | **-10.2964^*^** | **2.97346** | **.050** | **-20.5879** | **-.0050** |
|  | **Kempston** | **1.1457** | **2.91325** | **1.000** | **-8.9374** | **11.2288** |
|  | **Kentford** | **-3.5946** | **2.78398** | **.998** | **-13.2303** | **6.0411** |
|  | **Lent Rise** | **5.5183** | **3.01115** | **.921** | **-4.9036** | **15.9403** |
|  | Purfleet | 19.7129^*^ | 3.80166 | <.001 | 6.5550 | 32.8709 |
|  | **Redhill (B. Gravel)** | **9.6545** | **3.86342** | **.503** | **-3.7173** | **23.0262** |
|  | Stoke Newington | 14.6810^*^ | 2.35054 | <.001 | 6.5455 | 22.8165 |
|  | **Warsash** | **1.1388** | **3.25362** | **1.000** | **-10.1223** | **12.4000** |
| Furze Platt | Baker's Farm | -8.4776^*^ | 1.89890 | .001 | -15.0499 | -1.9053 |
|  | Barnham Heath | -27.0462^*^ | 1.83554 | <.001 | -33.3992 | -20.6932 |
|  | **Biddenham** | **-1.0999** | **1.62377** | **1.000** | **-6.7200** | **4.5201** |
|  | Cuxton 1-6 | 12.0903^*^ | 2.43213 | <.001 | 3.6724 | 20.5082 |
|  | Cuxton 7+ | 21.9433^*^ | 2.24590 | <.001 | 14.1700 | 29.7166 |
|  | Cuxton Tester | 13.0147^*^ | 1.68870 | <.001 | 7.1699 | 18.8595 |
|  | **Dunbridge** | **-7.9852** | **2.47716** | **.102** | **-16.5589** | **.5885** |
|  | Globe Pit | 25.4218^*^ | 1.58545 | <.001 | 19.9343 | 30.9092 |
|  | Groveland's Pit | -18.2816^*^ | 2.44084 | <.001 | -26.7296 | -9.8336 |
|  | **Kempston** | **-6.8395** | **2.36713** | **.240** | **-15.0324** | **1.3534** |
|  | Kentford | -11.5798^*^ | 2.20609 | <.001 | -19.2153 | -3.9443 |
|  | **Lent Rise** | **-2.4669** | **2.48662** | **1.000** | **-11.0733** | **6.1396** |
|  | **Purfleet** | **11.7277** | **3.40130** | **.052** | **-.0445** | **23.5000** |
|  | **Redhill (B. Gravel)** | **1.6693** | **3.47020** | **1.000** | **-10.3415** | **13.6801** |
|  | **Stoke Newington** | **6.6959^*^** | **1.62521** | **.004** | **1.0708** | **12.3209** |
|  | **Warsash** | **-6.8464** | **2.77530** | **.527** | **-16.4520** | **2.7593** |
| Globe Pit | Baker's Farm | -33.8993^*^ | 1.69319 | <.001 | -39.7596 | -28.0390 |
|  | Barnham Heath | -52.4679^*^ | 1.62182 | <.001 | -58.0812 | -46.8546 |
|  | Biddenham | -26.5217^*^ | 1.37757 | <.001 | -31.2896 | -21.7538 |
|  | Cuxton 1-6 | -13.3314^*^ | 2.27515 | <.001 | -21.2060 | -5.4569 |
|  | **Cuxton 7+** | **-3.4785** | **2.07488** | **.963** | **-10.6598** | **3.7029** |
|  | Cuxton Tester | -12.4071^*^ | 1.45354 | <.001 | -17.4379 | -7.3762 |
|  | Dunbridge | -33.4069^*^ | 2.32323 | <.001 | -41.4479 | -25.3660 |
|  | Furze Platt | -25.4218^*^ | 1.58545 | <.001 | -30.9092 | -19.9343 |
|  | Groveland's Pit | -43.7034^*^ | 2.28446 | <.001 | -51.6102 | -35.7966 |
|  | Kempston | -32.2613^*^ | 2.20554 | <.001 | -39.8949 | -24.6277 |
|  | Kentford | -37.0015^*^ | 2.03172 | <.001 | -44.0336 | -29.9695 |
|  | Lent Rise | -27.8886^*^ | 2.33332 | <.001 | -35.9645 | -19.8127 |
|  | **Purfleet** | **-13.6940^*^** | **3.29089** | **.004** | **-25.0841** | **-2.3039** |
|  | Redhill (B. Gravel) | -23.7524^*^ | 3.36205 | <.001 | -35.3889 | -12.1160 |
|  | Stoke Newington | -18.7259^*^ | 1.37927 | <.001 | -23.4997 | -13.9521 |
|  | Warsash | -32.2681^*^ | 2.63882 | <.001 | -41.4014 | -23.1349 |
| Groveland's Pit | **Baker's Farm** | **9.8041^*^** | **2.51216** | **.011** | **1.1092** | **18.4989** |
|  | **Barnham Heath** | **-8.7646^*^** | **2.46462** | **.037** | **-17.2949** | **-.2342** |
|  | Biddenham | 17.1817^*^ | 2.31122 | <.001 | 9.1823 | 25.1811 |
|  | Cuxton 1-6 | 30.3719^*^ | 2.93604 | <.001 | 20.2099 | 40.5339 |
|  | Cuxton 7+ | 40.2249^*^ | 2.78373 | <.001 | 30.5901 | 49.8597 |
|  | Cuxton Tester | 31.2963^*^ | 2.35729 | <.001 | 23.1374 | 39.4552 |
|  | **Dunbridge** | **10.2964^*^** | **2.97346** | **.050** | **.0050** | **20.5879** |
|  | Furze Platt | 18.2816^*^ | 2.44084 | <.001 | 9.8336 | 26.7296 |
|  | Globe Pit | 43.7034^*^ | 2.28446 | <.001 | 35.7966 | 51.6102 |
|  | **Kempston** | **11.4421^*^** | **2.88244** | **.008** | **1.4656** | **21.4185** |
|  | **Kentford** | **6.7018** | **2.75172** | **.551** | **-2.8222** | **16.2258** |
|  | Lent Rise | 15.8148^*^ | 2.98134 | <.001 | 5.4960 | 26.1335 |
|  | Purfleet | 30.0094^*^ | 3.77809 | <.001 | 16.9330 | 43.0858 |
|  | Redhill (B. Gravel) | 19.9509^*^ | 3.84024 | <.001 | 6.6594 | 33.2424 |
|  | Stoke Newington | 24.9775^*^ | 2.31223 | <.001 | 16.9746 | 32.9804 |
|  | **Warsash** | **11.4352^*^** | **3.22605** | **.038** | **.2695** | **22.6010** |
| Kempston | **Baker's Farm** | **-1.6380** | **2.44060** | **1.000** | **-10.0852** | **6.8092** |
|  | Barnham Heath | -20.2066^*^ | 2.39164 | <.001 | -28.4844 | -11.9289 |
|  | **Biddenham** | **5.7396** | **2.23324** | **.449** | **-1.9899** | **13.4691** |
|  | Cuxton 1-6 | 18.9298^*^ | 2.87506 | <.001 | 8.9789 | 28.8808 |
|  | Cuxton 7+ | 28.7828^*^ | 2.71933 | <.001 | 19.3709 | 38.1948 |
|  | Cuxton Tester | 19.8542^*^ | 2.28088 | <.001 | 11.9598 | 27.7486 |
|  | **Dunbridge** | **-1.1457** | **2.91325** | **1.000** | **-11.2288** | **8.9374** |
|  | **Furze Platt** | **6.8395** | **2.36713** | **.240** | **-1.3534** | **15.0324** |
|  | Globe Pit | 32.2613^*^ | 2.20554 | <.001 | 24.6277 | 39.8949 |
|  | **Groveland's Pit** | **-11.4421^*^** | **2.88244** | **.008** | **-21.4185** | **-1.4656** |
|  | **Kentford** | **-4.7403** | **2.68655** | **.942** | **-14.0387** | **4.5582** |
|  | **Lent Rise** | **4.3727** | **2.92131** | **.988** | **-5.7383** | **14.4836** |
|  | Purfleet | 18.5673^*^ | 3.73090 | <.001 | 5.6542 | 31.4803 |
|  | **Redhill (B. Gravel)** | **8.5088** | **3.79382** | **.697** | **-4.6220** | **21.6397** |
|  | Stoke Newington | 13.5354^*^ | 2.23429 | <.001 | 5.8023 | 21.2685 |
|  | **Warsash** | **-.0068** | **3.17065** | **1.000** | **-10.9808** | **10.9671** |
| Kentford | **Baker's Farm** | **3.1022** | **2.28474** | **.996** | **-4.8055** | **11.0100** |
|  | Barnham Heath | -15.4664^*^ | 2.23237 | <.001 | -23.1929 | -7.7399 |
|  | Biddenham | 10.4798^*^ | 2.06176 | <.001 | 3.3439 | 17.6158 |
|  | Cuxton 1-6 | 23.6701^*^ | 2.74399 | <.001 | 14.1728 | 33.1674 |
|  | Cuxton 7+ | 33.5231^*^ | 2.58037 | <.001 | 24.5921 | 42.4540 |
|  | Cuxton Tester | 24.5945^*^ | 2.11328 | <.001 | 17.2802 | 31.9088 |
|  | **Dunbridge** | **3.5946** | **2.78398** | **.998** | **-6.0411** | **13.2303** |
|  | Furze Platt | 11.5798^*^ | 2.20609 | <.001 | 3.9443 | 19.2153 |
|  | Globe Pit | 37.0015^*^ | 2.03172 | <.001 | 29.9695 | 44.0336 |
|  | **Groveland's Pit** | **-6.7018** | **2.75172** | **.551** | **-16.2258** | **2.8222** |
|  | **Kempston** | **4.7403** | **2.68655** | **.942** | **-4.5582** | **14.0387** |
|  | **Lent Rise** | **9.1129** | **2.79241** | **.091** | **-.5519** | **18.7778** |
|  | Purfleet | 23.3075^*^ | 3.63085 | <.001 | 10.7407 | 35.8743 |
|  | **Redhill (B. Gravel)** | **13.2491^*^** | **3.69548** | **.033** | **.4586** | **26.0396** |
|  | Stoke Newington | 18.2757^*^ | 2.06290 | <.001 | 11.1357 | 25.4156 |
|  | **Warsash** | **4.7334** | **3.05230** | **.982** | **-5.8309** | **15.2978** |
| Lent Rise | **Baker's Farm** | **-6.0107** | **2.55666** | **.616** | **-14.8596** | **2.8382** |
|  | Barnham Heath | -24.5793^*^ | 2.50996 | <.001 | -33.2666 | -15.8920 |
|  | **Biddenham** | **1.3669** | **2.35952** | **1.000** | **-6.7997** | **9.5335** |
|  | Cuxton 1-6 | 14.5572^*^ | 2.97421 | <.001 | 4.2631 | 24.8513 |
|  | Cuxton 7+ | 24.4102^*^ | 2.82396 | <.001 | 14.6361 | 34.1842 |
|  | Cuxton Tester | 15.4815^*^ | 2.40467 | <.001 | 7.1587 | 23.8044 |
|  | **Dunbridge** | **-5.5183** | **3.01115** | **.921** | **-15.9403** | **4.9036** |
|  | **Furze Platt** | **2.4669** | **2.48662** | **1.000** | **-6.1396** | **11.0733** |
|  | Globe Pit | 27.8886^*^ | 2.33332 | <.001 | 19.8127 | 35.9645 |
|  | Groveland's Pit | -15.8148^*^ | 2.98134 | <.001 | -26.1335 | -5.4960 |
|  | **Kempston** | **-4.3727** | **2.92131** | **.988** | **-14.4836** | **5.7383** |
|  | **Kentford** | **-9.1129** | **2.79241** | **.091** | **-18.7778** | **.5519** |
|  | **Purfleet** | **14.1946^*^** | **3.80783** | **.020** | **1.0153** | **27.3739** |
|  | **Redhill (B. Gravel)** | **4.1362** | **3.86950** | **1.000** | **-9.2566** | **17.5289** |
|  | **Stoke Newington** | **9.1627^*^** | **2.36051** | **.011** | **.9927** | **17.3327** |
|  | **Warsash** | **-4.3795** | **3.26083** | **.996** | **-15.6656** | **6.9066** |
| Purfleet | Baker's Farm | -20.2053^*^ | 3.45284 | <.001 | -32.1560 | -8.2546 |
|  | Barnham Heath | -38.7739^*^ | 3.41840 | <.001 | -50.6054 | -26.9424 |
|  | **Biddenham** | **-12.8277^*^** | **3.30951** | **.012** | **-24.2823** | **-1.3731** |
|  | **Cuxton 1-6** | **.3626** | **3.77247** | **1.000** | **-12.6944** | **13.4195** |
|  | **Cuxton 7+** | **10.2156** | **3.65518** | **.295** | **-2.4354** | **22.8665** |
|  | **Cuxton Tester** | **1.2869** | **3.34185** | **1.000** | **-10.2796** | **12.8535** |
|  | Dunbridge | -19.7129^*^ | 3.80166 | <.001 | -32.8709 | -6.5550 |
|  | **Furze Platt** | **-11.7277** | **3.40130** | **.052** | **-23.5000** | **.0445** |
|  | **Globe Pit** | **13.6940^*^** | **3.29089** | **.004** | **2.3039** | **25.0841** |
|  | Groveland's Pit | -30.0094^*^ | 3.77809 | <.001 | -43.0858 | -16.9330 |
|  | Kempston | -18.5673^*^ | 3.73090 | <.001 | -31.4803 | -5.6542 |
|  | Kentford | -23.3075^*^ | 3.63085 | <.001 | -35.8743 | -10.7407 |
|  | **Lent Rise** | **-14.1946^*^** | **3.80783** | **.020** | **-27.3739** | **-1.0153** |
|  | **Redhill (B. Gravel)** | **-10.0584** | **4.51205** | **.706** | **-25.6752** | **5.5583** |
|  | **Stoke Newington** | **-5.0319** | **3.31022** | **.985** | **-16.4890** | **6.4252** |
|  | Warsash | -18.5741^*^ | 4.00232 | <.001 | -32.4266 | -4.7216 |
| Redhill (B. Gravel) | **Baker's Farm** | **-10.1469** | **3.52073** | **.244** | **-22.3325** | **2.0388** |
|  | Barnham Heath | -28.7155^*^ | 3.48697 | <.001 | -40.7843 | -16.6467 |
|  | **Biddenham** | **-2.7693** | **3.38029** | **1.000** | **-14.4688** | **8.9303** |
|  | **Cuxton 1-6** | **10.4210** | **3.83470** | **.344** | **-2.8513** | **23.6934** |
|  | Cuxton 7+ | 20.2740^*^ | 3.71938 | <.001 | 7.4008 | 33.1472 |
|  | **Cuxton Tester** | **11.3454** | **3.41195** | **.076** | **-.4638** | **23.1545** |
|  | **Dunbridge** | **-9.6545** | **3.86342** | **.503** | **-23.0262** | **3.7173** |
|  | **Furze Platt** | **-1.6693** | **3.47020** | **1.000** | **-13.6801** | **10.3415** |
|  | Globe Pit | 23.7524^*^ | 3.36205 | <.001 | 12.1160 | 35.3889 |
|  | Groveland's Pit | -19.9509^*^ | 3.84024 | <.001 | -33.2424 | -6.6594 |
|  | **Kempston** | **-8.5088** | **3.79382** | **.697** | **-21.6397** | **4.6220** |
|  | **Kentford** | **-13.2491^*^** | **3.69548** | **.033** | **-26.0396** | **-.4586** |
|  | **Lent Rise** | **-4.1362** | **3.86950** | **1.000** | **-17.5289** | **9.2566** |
|  | **Purfleet** | **10.0584** | **4.51205** | **.706** | **-5.5583** | **25.6752** |
|  | **Stoke Newington** | **5.0265** | **3.38098** | **.988** | **-6.6754** | **16.7285** |
|  | **Warsash** | **-8.5157** | **4.06104** | **.794** | **-22.5714** | **5.5400** |
| Stoke Newington | Baker's Farm | -15.1734^*^ | 1.73047 | <.001 | -21.1628 | -9.1841 |
|  | Barnham Heath | -33.7420^*^ | 1.66070 | <.001 | -39.4899 | -27.9941 |
|  | Biddenham | -7.7958^*^ | 1.42315 | <.001 | -12.7215 | -2.8701 |
|  | **Cuxton 1-6** | **5.3945** | **2.30303** | **.623** | **-2.5766** | **13.3655** |
|  | Cuxton 7+ | 15.2474^*^ | 2.10541 | <.001 | 7.9604 | 22.5345 |
|  | **Cuxton Tester** | **6.3188^*^** | **1.49681** | **.003** | **1.1382** | **11.4994** |
|  | Dunbridge | -14.6810^*^ | 2.35054 | <.001 | -22.8165 | -6.5455 |
|  | **Furze Platt** | **-6.6959^*^** | **1.62521** | **.004** | **-12.3209** | **-1.0708** |
|  | Globe Pit | 18.7259^*^ | 1.37927 | <.001 | 13.9521 | 23.4997 |
|  | Groveland's Pit | -24.9775^*^ | 2.31223 | <.001 | -32.9804 | -16.9746 |
|  | Kempston | -13.5354^*^ | 2.23429 | <.001 | -21.2685 | -5.8023 |
|  | Kentford | -18.2757^*^ | 2.06290 | <.001 | -25.4156 | -11.1357 |
|  | **Lent Rise** | **-9.1627^*^** | **2.36051** | **.011** | **-17.3327** | **-.9927** |
|  | **Purfleet** | **5.0319** | **3.31022** | **.985** | **-6.4252** | **16.4890** |
|  | **Redhill (B. Gravel)** | **-5.0265** | **3.38098** | **.988** | **-16.7285** | **6.6754** |
|  | Warsash | -13.5422^*^ | 2.66290 | <.001 | -22.7588 | -4.3256 |
| Warsash | **Baker's Farm** | **-1.6312** | **2.83822** | **1.000** | **-11.4546** | **8.1922** |
|  | Barnham Heath | -20.1998^*^ | 2.79623 | <.001 | -29.8779 | -10.5217 |
|  | **Biddenham** | **5.7464** | **2.66202** | **.755** | **-3.4671** | **14.9600** |
|  | Cuxton 1-6 | 18.9367^*^ | 3.21946 | <.001 | 7.7937 | 30.0796 |
|  | Cuxton 7+ | 28.7897^*^ | 3.08119 | <.001 | 18.1253 | 39.4540 |
|  | Cuxton Tester | 19.8611^*^ | 2.70211 | <.001 | 10.5087 | 29.2134 |
|  | **Dunbridge** | **-1.1388** | **3.25362** | **1.000** | **-12.4000** | **10.1223** |
|  | **Furze Platt** | **6.8464** | **2.77530** | **.527** | **-2.7593** | **16.4520** |
|  | Globe Pit | 32.2681^*^ | 2.63882 | <.001 | 23.1349 | 41.4014 |
|  | **Groveland's Pit** | **-11.4352^*^** | **3.22605** | **.038** | **-22.6010** | **-.2695** |
|  | **Kempston** | **.0068** | **3.17065** | **1.000** | **-10.9671** | **10.9808** |
|  | **Kentford** | **-4.7334** | **3.05230** | **.982** | **-15.2978** | **5.8309** |
|  | **Lent Rise** | **4.3795** | **3.26083** | **.996** | **-6.9066** | **15.6656** |
|  | Purfleet | 18.5741^*^ | 4.00232 | <.001 | 4.7216 | 32.4266 |
|  | **Redhill (B. Gravel)** | **8.5157** | **4.06104** | **.794** | **-5.5400** | **22.5714** |
|  | Stoke Newington | 13.5422^*^ | 2.66290 | <.001 | 4.3256 | 22.7588 |
| Based on observed means.  The error term is Mean Square(Error) = 437.473. | | | | | | |
| *. The mean difference is significant at the 0.05 level. | | | | | | |

A cluster analysis using length, width, thickness and elongation of flakes aligns with previous observations demonstrating links between excavated and well collected sites.


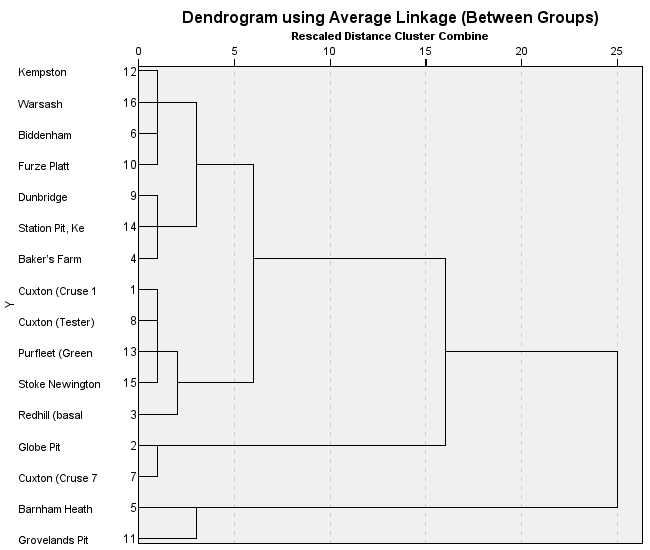

Supplement: Supplementary file 3 — Supplementary file3 (DOCX 118 KB) [file 41982_2025_217_MOESM3_ESM.docx]
